# Physiological status of plant tissue affects the frequency and types of mutations induced by carbon-ion irradiation in Arabidopsis

**DOI:** 10.1038/s41598-018-19278-1

**Published:** 2018-01-23

**Authors:** Yoshihiro Hase, Katsuya Satoh, Satoshi Kitamura, Yutaka Oono

**Affiliations:** Takasaki Advanced Radiation Research Institute, National Institutes for Quantum and Radiological Science and Technology (QST), 1233 Watanuki, Takasaki, Gunma 370-1292 Japan

## Abstract

Ionizing radiation including heavy-ion beams has been widely used in mutation breeding. Dry seeds, seedlings, and cultured tissues are often used for mutagenesis; however, little is known about the differences in induced mutations among them. Here, we examined the characteristics of mutations using randomly chosen Arabidopsis M_2_ plants derived from dry seeds and seedlings irradiated with carbon ions. The mutation frequency was 1.4–1.9 times higher in dry-seed irradiation than in seedling irradiation. This difference was mainly due to the three-times higher frequency of insertions and deletions (InDels) in dry-seed irradiation than in seedling irradiation. This difference increased the proportion of mutations predicted to affect gene function among all mutations identified by whole genome re-sequencing. Our results demonstrate that the physiological status of plant tissue greatly affects the characteristics of mutations induced by ionizing radiation, and that dry seeds are more suitable materials than seedlings for inducing loss-of-function mutations. The results also showed that single base deletions often occurred in homopolymeric sequences, while InDels larger than 2–3 bp often occurred in or near polynucleotide-repeat or microhomologous sequences. Interestingly, microhomology was less commonly found around large deletions (≥50 bp), suggesting that the rejoining process differs depending on the deletion size.

## Introduction

Mutagenesis with heavy-ion beams, which are characterized by their high linear energy transfer (LET), is an effective means to generate new plant varieties, and there is increasing evidence that ion beams induce mutations more efficiently than do gamma-rays or X-rays^1,2^. Many studies have focused on the characteristics of mutations induced by ion beams, with an aim to develop more efficient mutagenesis techniques. Most analyses of mutations have focused on those in specific genes through phenotype-based screening^3–8^. However, those approaches take time and effort, and there is some bias due to the genes of interest and the essential genes nearby. Recently, genome wide analysis using next generation sequencing technology has rapidly advanced our understanding of the characteristics of mutations induced by ionizing radiation. Mutations induced by fast neutrons have been reported in Arabidopsis^9^ and rice^10^. Structural alterations in chromosomes induced by high-LET argon ions have been thoroughly examined in Arabidopsis^11^. However, further studies are required to obtain a comprehensive view of the characteristics of mutations induced by high-LET radiation.

Okamura *et al*.^12^ reported that more chrysanthemum flower colour mutants were obtained from cultured petals than from leaves irradiated by ion beam irradiation, whereas the regeneration frequency and the stem length of the regenerated plants did not differ between these two tissues. Similarly, compared with mock-treated petunia seedlings, those treated with sucrose (to stimulate colour pigment biosynthesis) yielded a higher frequency of flower-colour mutants after ion beam irradiation, although the frequency of chlorophyll mutants was unaffected^13^. These results strongly suggest that physiological status of plant tissue is an important factor in efficient mutagenesis. In fact, many types of plant tissues, i.e., dry seeds, seedlings, lateral buds, and callus are treated with ionizing radiation in mutation breeding. Radiation sensitivity differs greatly among different tissues, for example, dry seeds are much less sensitive than living tissues. However, the effects of physiological status of plant tissue on the characteristics of mutations are largely unknown. In this study, we considered the effects of physiological status of plant tissue on the frequency and types of mutations induced by carbon ion irradiation in Arabidopsis. Dry seeds and seedlings, which had quite different moisture contents and metabolic activities, were irradiated with the same effective dose as judged by survival rate, and randomly chosen plants in the following generation were subjected to whole genome re-sequencing. The results showed that dry-seed irradiation gave a higher mutation frequency than did seedling irradiation, and this difference was mostly caused by the increased frequency of InDels in dry-seed irradiation. The obtained data were compared and discussed in the context of results from other studies on fast neutron mutation and classical phenotype-based analyses. This study provides strong evidence for the first time that the physiological status of plant tissue greatly affects the frequency and types of mutations at the molecular level.

## Results

### Effects of carbon ion irradiation on plant growth

**Figure 1:**
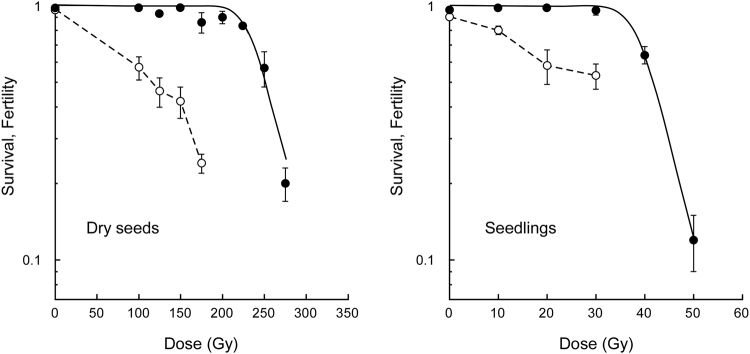
Dose–response relationships for survival rate and fertility of Arabidopsis plants derived from dry seeds and 7-day-old seedlings irradiated with carbon ions. Closed and open circles represent survival rate and fertility, respectively. Survival curves were drawn on the basis of the single hit-multitarget theory as previously described^5^. Fertility represents ratio of the number of fertilized ovules to the total number of ovules. Data points are mean ± standard error of three replications with 25 plants for survival, and mean ± standard error of more than 50 siliques for fertility.

Since the radiation sensitivity is quite different between dry seeds and seedlings, the characteristics of mutations were compared with the same effective dose based on the survival rate. Figure 1 shows the dose–response curves for survival rate and fertility. The dose corresponding to the shoulder of the survival curve (*Dq*) was 241 Gy for dry-seed irradiation and 41 Gy for seedling irradiation. Thus, the 7-day-old seedlings were six times more sensitive than the dry seeds to carbon ion irradiation. The fertility decreased as the dose increased in both materials, but the reduction in fertility relative to the survival rate was more marked in irradiated dry seeds. Doses corresponding to 50% and 75% of *Dq* for each material were used to compare the characteristics of mutations; 125 Gy (fertility at this dose; 46 ± 6%) and 175 Gy (24 ± 2%), respectively, for dry-seed irradiation, and 20 Gy (58 ± 9%) and 30 Gy (53 ± 6%), respectively, for seedling irradiation. Progeny seeds were obtained from individual plants in each of the four experimental groups by self-pollination.

### Detection of mutations by whole genome re-sequencing

As described in the materials and methods section, whole genome re-sequencing was conducted using six randomly chosen M_2_ plants from each of the four experimental groups. Approximately four gigabases of sequencing data per sample were mapped to the reference genome (Table S1). The average depth was about 33× and more than 98% of the genome was covered at 10× or more on average. The identified mutations were classified into seven categories; (1) Single base substitution (SBS); (2) single base deletion (−1); (3) deletion of two or more bp (Del_≥2 bp); (4) single base insertion (+1); (5) insertion of two or more bp (Ins_≥2 bp); (6) complex type; and (7) structural variation (SV). A few deletion mutations were accompanied by an insertion of unknown sequence. In those cases, the length of the sequence alteration represented the difference from the reference sequence, e.g., a putative 10-base deletion accompanied by a 3-base insertion of unknown sequence was considered as a 7-base deletion. More than two consecutive SBSs were regarded as a single mutation event and were classified as the complex type, because their generation mechanism may differ from that of a single isolated SBS. More than two SBSs and/ or short insertion and deletions (<50 bp) identified with a gap of less than 10 bases were also regarded as a single mutation event and were classified as the complex type. In those cases, at least two bases identical to the reference genome were assumed to be a non-mutated sequence.

To verify the accuracy and reliability of the data, the sequences of 37 candidate mutations (10 SBSs, eight −1, seven Del_≥2 bp, three +1, five Ins_≥2 bp, and four complex-type) were confirmed by Sanger sequencing (Table S2). The sequence of wild-type DNA was identical to the reference sequence at all tested sites. Two of the 37 candidate mutations; a heterozygous single base deletion of A (sample 175-1-4, chr5 20,942,238) and a heterozygous two-base AT insertion (sample 20-11-2, chr5 638,204), were not identified in the corresponding M_2_ plant DNA, and were therefore deemed as false positives. The allele frequency (AF) of these two candidate mutations was relatively low (0.25 and 0.22, respectively). However, another candidate mutation with AF 0.23 was confirmed to be true (125-10-5, chr2 13,338,270, heterozygous single base deletion of A). These observations suggested that the candidate mutations with AF of 0.25 or lower contained some false positives. To ensure the reliability of the results, candidate mutations with AF of 0.25 or lower were excluded from this study. The reliability of this method is supported by the fact that the AF of heterozygous mutations showed a bilaterally symmetrical distribution with a peak around 0.5 (the theoretically expected AF for heterozygous mutations), after removal of candidate mutations with AF of 0.25 or lower (Fig. S1). The SVs were also detected in this study and most of the junction points were confirmed by the Integrative Genomics Viewer (IGV) (Table S3, Fig. S2). However, since the short-read sequencing technology has a limited ability to evaluate the frequency and detailed structure of the SVs, the data of SVs were excluded from a comparison of the characteristics of mutations.

### Dry-seed irradiation induces more deletions than seedling irradiation

**Figure 2:**
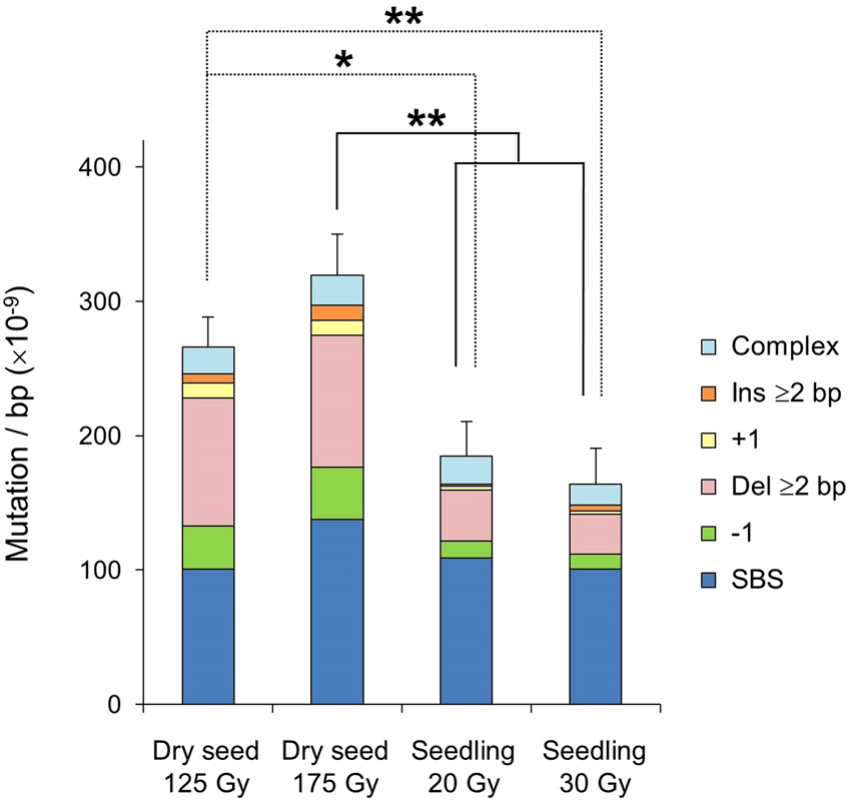
Mutation frequency and types of mutations induced by carbon-ion irradiation of dry seeds and seedlings. Mutation frequency was calculated as total number of mutation events divided by length of reference genome 119,146,348 bp (TAIR10.27). Data are mean of six M_2_ plants for each experimental group. Error bars represent standard errors for total mutation frequency. Asterisks indicate significant difference in total mutation frequency (*t*-test, *p < 0.05 and **p < 0.01).

In total, 667 mutations were identified in this study (Table 1). The number of mutations per sample ranged from 20 to 39 and 27 to 50 in dry seed_125 Gy and 175 Gy, respectively, and 11 to 33 and 12 to 33 in seedling_20 Gy and 30 Gy, respectively (Table S4). The mutation frequencies (MFs) were calculated as total number of mutation events divided by length of reference genome. The mean MFs were 2.7 and 3.2 (×10^−7^/bp) in dry seed_125 Gy and 175 Gy, respectively, and 1.8 and 1.6 (×10^−7^/bp) in seedling_20 Gy and 30 Gy, respectively (Fig. 2). The MF was significantly higher in dry-seed irradiation than in seedling irradiation. The MF did not show a dose–response relationship. The biggest difference between dry-seed irradiation and seedling irradiation was the types of mutations. The frequency of SBS did not greatly differ between the two materials (dry seed; 1.0 and 1.4 (×10^−7^/bp), seedling: 1.1 and 1.0 (×10^−7^/bp)). However, the frequency of deletions including −1 and Del_≥2 bp was approximately three times higher in dry-seed irradiation than in seedling irradiation (dry seed; 1.3 and 1.4 (×10^−7^/bp), seedling; 0.5 and 0.4 (×10^−7^/bp). Corresponding to this difference, the proportion of deletion mutations out of total mutations was 48% and 43% in dry-seed irradiation, and 27% and 25% in seedling irradiation (Table 1). The frequency of insertion mutations also tended to be higher in dry-seed irradiation than in seedling irradiation (dry seed; 1.8 and 2.2 (×10^−8^/bp), seedling; 0.4 and 0.7 (×10^−8^/bp)). Thus, the increased frequency of InDel mutations largely accounted for the higher MF in dry-seed irradiation.

**Table 1:** Summary of identified mutations.

		Dry seed 125 Gy	Dry seed 175 Gy	Seedling 20 Gy	Seedling 30 Gy
Single base substitution		72 (38%)	98 (43%)	78 (59%)	72 (62%)
Deletion	Single base	23	28	9	8
	≥2 bp	68	70	27	21
	Total	91 (48%)	98 (43%)	36 (27%)	29 (25%)
Insertion	Single base	8	8	2	2
	≥2 bp	5	8	1	3
	Total	13 (7%)	16 (7%)	3 (2%)	5 (4%)
Complex type		14 (7%)	16 (7%)	15 (11%)	11 (9%)
Grand total		190	228	132	117

Data for each experimental group was obtained from 6 independent plants. Numbers in parentheses represent percentage of total mutations for each group.

### SBSs and InDels induced by carbon-ion irradiation

**Figure 3:**
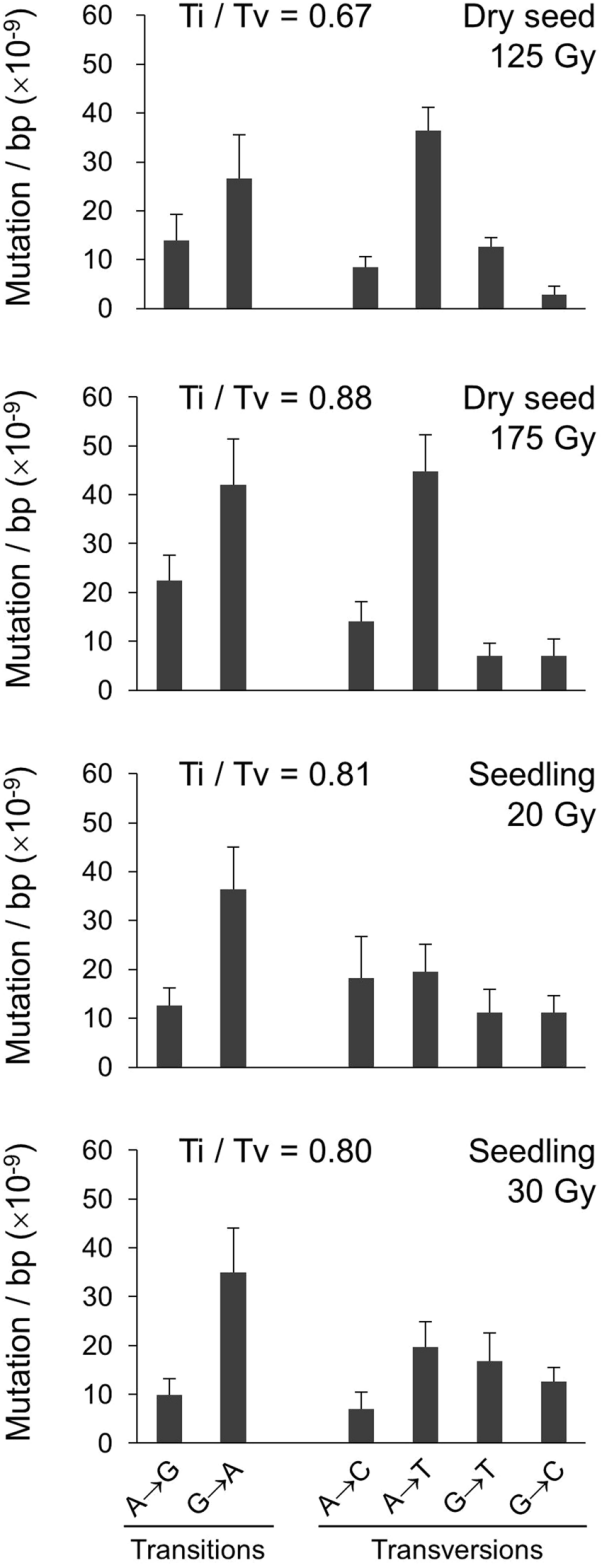
Frequency and spectra of single base substitutions. Complementary substitutions (e.g. G to A and C to T) are merged. Ti/Tv: ratio of total transitions to transversions.

The frequency and spectra of SBSs are shown in Fig. 3. The G to A transition was the main SBS in all experimental groups. The A to T transversion was one of the main mutations in dry-seed irradiation but not in seedling irradiation. The proportions of the six kinds of SBS differed significantly between the two materials, mainly because of the ratio of the A to T transversion (Chi-squared test, *p* < 0.01). The ratio of transition to transversion (Ti/Tv) ranged from 0.67 to 0.88 and did not differ significantly among the experimental groups (Chi-squared test).

**Figure 4:**
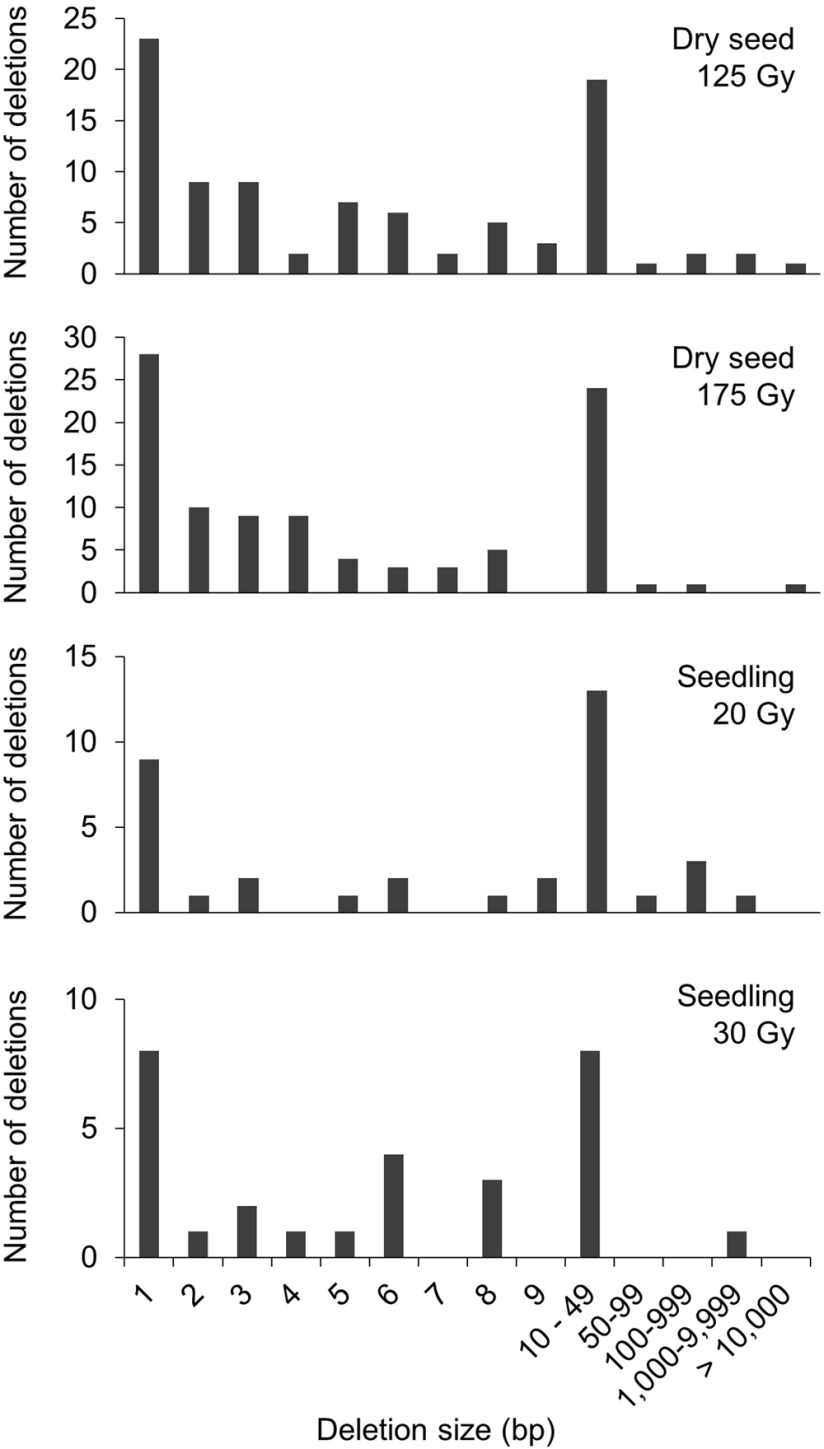
Distribution of deletion sizes.

The distribution of deletion size is shown in Fig. 4. Single base deletions were the most common in all experimental groups. The number of mutations tended to decrease as the deletion size increased, and most were smaller than 50 bp (dry seed; 93% and 97%, seedling: 86% and 97%). There was no significant difference in the distribution of deletion size among the experimental groups (Chi-squared test).

### Sequence characteristics of InDel and complex-type mutation sites

Homopolymeric sequences (≥3 bases, e.g. AAAA) were often found at the sites of single base deletions (22/49; 45%), while polynucleotide repeats (e.g. TATA) were less common (9/49; 18%) (Table 2). Single base deletions occurred more often at A/T sites (33/49; 67%) than at G/C sites in dry-seed irradiation (Table S5). Similar trends in single base deletions were observed in irradiated seedlings. The features of Del_≥2 bp mutation sites differed from those of single base deletions: Polynucleotide repeats at the mutation site or microhomology at the rejoined site were often detected (63/129; 49%), but homopolymeric sequences, which were often observed around single base deletions, were rare (12/129; 9%) in irradiated dry seeds (Table 2). Similar trends were observed in Del_≥2 bp sites in irradiated seedlings. Interestingly, the frequency of polynucleotide repeats or microhomology at the mutation site differed depending on the deletion size. The frequency of deletions with polynucleotide repeats or microhomology was 48% (38/80) in 2–9-base deletions, 58% (23/40) in 10–49-base deletions, and 22% (2/9) in ≥50-base deletions in dry-seed irradiation. In seedling irradiation, the frequency was 42% (8/19) in 2–9-base deletions, 53% (10/19) in 10–49-base deletions, and 17% (1/6) in ≥50-base deletions. Thus, polynucleotide repeats and microhomology were less frequently found around deletions of ≥50 bp.

**Table 2:** Characteristics of sequence at mutation sites or rejoined sites of insertion and deletion mutations.

Type of Mutation	Irradiation material	Homopolymeric sequence	Polynucleotide repeat or microhomology		
			Total	Breakdown by length	
−1	Dry seed	45% (22/49)	18% (9/49)		
	Seedling	50% (8/16)	6% (1/16)		
Del_≥2 bp	Dry seed	9% (12/129)	49% (63/129)	2–9 bp	48% (38/80)
				10–49 bp	58% (23/40)
				≥50 bp	22% (2/9)
	Seedling	5% (2/44)	43% (19/44)	2–9 bp	42% (8/19)
				10–49 bp	53% (10/19)
				≥50 bp	17% (1/6)
+1	Combined*	44% (8/18)	27% (5/18)		
Ins_≥2 bp	Combined*	29% (5/17)	76% (13/17)		

Numbers in parentheses represent number of mutation events associated with homopolymeric sequences (≥3 bp, e.g. AAAA), polynucleotide repeats (e.g. TATA) or microhomology at mutation site or rejoined site/ total number of mutation events corresponding to each category. Details of sequences are shown in Table S5.

*Combined data from dry-seed irradiation and seedling irradiation.

Single base insertions were mainly at A/T sites; 93% (17/18) in the combined data of dry-seed irradiation and seedling irradiation (Table S5). Homopolymeric sequences were found more often (8/18; 44%) than polynucleotide repeats (5/18; 27%) (Table 2). For Ins_≥2 bp, 13 out of 17 mutations (76%) contained a polynucleotide repeat or microhomology at the rejoined site, while homopolymeric sequences were less common (5/17; 29%).

There were no significant differences in the frequency and sequences of complex-type mutations between dry-seed irradiation and seedling irradiation (Table S6).

### Zygosity and number of genes affected

The theoretically expected ratio of heterozygous to homozygous mutations was 2.0, because M_2_ plants were examined in this study. The actual ratio for each sample varied from 0.6 to 11.0 (Table S4). Particularly in sample 30-7-1, all 33 identified mutations were heterozygous. The mean ratios of heterozygous to homozygous mutations were 3.3 ± 1.2 and 2.5 ± 0.7 in dry seed_125 Gy and 175 Gy, respectively, and 3.6 ± 1.4 and 4.6 ± 1.5 in seedling_20 Gy and 30 Gy, respectively. There was no significant difference in mean ratio among the four experimental groups and also no significant difference from the expected ratio of 2.0 (*t*-test).

The number of protein-coding genes with non-synonymous amino acid changes greatly varied among the samples, from 0 to 70, depending on the involvement of large deletions (Table 3, Fig. S3). Sixty genes were lost by a 283-kb deletion in sample 175-4-1 and 32 genes were lost by a 245-kb deletion in sample 125-6-1. The mean number of affected genes (in homozygous state) was 7.0 ± 5.3 and 13.3 ± 10.2 per sample in dry-seed irradiation, and 1.2 ± 0.5 and 0.5 ± 0.2 per sample in seedling irradiation (Table 3). We did not found apparent bias in the distribution of mutation events across chromosomes that resulted in non-synonymous amino acid changes (Fig. S3). The mean number of mutation events that resulted in amino acid changes in protein-coding genes was 6.3 ± 1.1 and 6.5 ± 1.8 per sample in dry seed_125 Gy and 175 Gy, respectively, and 4.5 ± 2.1 and 2.8 ± 1.0 per sample in seedling_20 Gy and 30 Gy, respectively (Table S7).

**Table 3:** Number of protein-coding genes with non-synonymous amino acid changes.

Dry seed 125 Gy							
Sample	125-10-5	125-12-1	125-2-2	125-4-1	125-5-1	125-6-1	Total
Homo	0	1	4	0	4	33^a^	42 (7.0 ± 5.3)
Hetero	7	11^b^	10	5	8	2	43
Total	7	12	14	5	12	35	85 (14.2 ± 4.4)
**Dry seed 175 Gy**							
**Sample**	**175-1-4**	**175-12-1**	**175-2-4**	**175-4-1**	**175-5-1**	**175-6-1**	**Total**
Homo	4	1	2	64^c^	7	2	80 (13.3 ± 10.2)
Hetero	2	2	9	6	6	13	38
Total	6	3	11	70	13	15	118 (19.7 ± 10.2)
**Seedling 20 Gy**							
**Sample**	**20-11-2**	**20-1-2**	**20-2-3**	**20-3-1**	**20-4-4**	**20-5-3**	**Total**
Homo	2	1	0	3	1	0	7 (1.2 ± 0.5)
Hetero	3	6	4	0	0	14	27
Total	5	7	4	3	1	14	34 (5.7 ± 1.9)
**Seedling 30 Gy**							
**Sample**	**30-11-2**	**30-2-1**	**30-3-4**	**30-4-1**	**30-6-2**	**30-7-1**	**Total**
Homo	0	1	1	1	0	0	3 (0.5 ± 0.2)
Hetero	0	4	2	3	1	7	17
Total	0	5	3	4	1	7	20 (3.3 ± 1.1)

Transposable elements, and pseudogenes are not included. Numbers in parentheses show mean ± standard error (6 samples per group).

^a^Thirty-two genes were involved in 245-kb deletion.

^b^Six genes were involved in 6.3-kb deletion.

^c^Sixty genes were involved in 283-kb deletion.

## Discussion

Dry seeds are much less sensitive than seedlings to ionizing radiation, mainly because of their lower water content. The biological effects of radiation result from direct and indirect actions of radiation, and a high moisture content results in increased indirect effects via radiolysis of water molecules. The reduction of fertility relative to survival rate was more marked in irradiated dry seeds than in irradiated seedlings (Fig. 1). This suggests that the shoot meristem of the plants derived from irradiated dry seeds contains more deleterious mutations, which lead to abnormal gametogenesis and embryonic lethality, compared with plants derived from seedlings irradiated at the same effective dose (as judged by survival reduction). This idea is consistent with the fact that the MF in the M_2_ generation was higher in dry-seed irradiation than in seedling irradiation (Fig. 2). Interestingly, the SBS frequency was similar between dry-seed irradiation and seedling irradiation; the three-times higher InDel frequency was the main reason for the higher MF in dry-seed irradiation than in seedling irradiation. These results could be attributed to the higher ratio of direct effects due to the low water content of dry seeds^14–16^. Alternatively, in the case of seedling irradiation, cells with InDels may have proliferated more slowly, resulting in their rapid elimination from the meristem (a tissue in which cells actively proliferate during and after irradiation). In fact, we demonstrated that the size of the mutant sector increased as the LET of ionizing radiation increased in Arabidopsis^4,5^. This observation suggests that some severely damaged cells are eliminated from the meristem. Differences in chromatin structure and/or repair processes may also be involved, because cells in dry seeds contain highly condensed chromatin^17^. From the viewpoint of practical mutation breeding, genetic alteration by a small number of mutations is preferable to minimize the number of accompanying undesirable mutations. The proportion of mutation events that resulted in non-synonymous amino acid changes was 26% (49/190) and 25% (59/228) in dry seed_125 Gy and 175 Gy, respectively, and 23% (34/132) and 17% (20/117) in seedling_20 Gy and 30 Gy, respectively, and this difference could be attributed to the higher ratio of InDels in dry-seed irradiation. This suggests that dry seeds are more efficient materials than seedlings for practical mutation breeding using ion beams.

The MF including both homozygous and heterozygous mutations induced by the carbon ions (surface LET, 107 keV/μm) was 2.7–3.2 × 10^−7^/bp in dry-seed irradiation, and 1.6–1.8 × 10^−7^/bp in seedling irradiation (Fig. 2). Assuming Mendelian inheritance, M_1_ plants were expected to contain 1.33 times more mutations than M_2_ plants. Therefore, the estimated MFs in the M_1_ generation ranged from 2.1 to 4.3 × 10^−7^/bp, 30–61 times higher than the spontaneous MF (7.1 × 10^−9^/bp) reported in Arabidopsis^18^. Fast neutron radiation is another type of high-LET radiation that has been used to induce mutations in plants. Mutations caused by dry-seed fast neutron irradiation have been detected by whole-genome sequencing in Arabidopsis^9^ and rice^10^. Belfield *et al*.^9^ examined six mutant lines in the M_3_ generation and reported that the estimated MF in the M_1_ generation was 3.6 × 10^−7^/bp. They observed 18 homozygous mutations per line on average in the M_3_ generation (assuming Mendelian inheritance, the expected number of homozygous mutations in the M_2_ generation was 12). These values are comparable to the MF and the number of homozygous mutations (10.5–13.8 in M_2_ generation, Table S4) observed in dry-seed irradiation in this study. Li *et al*.^10^ detected 59 homozygous mutations per plant on average by re-sequencing 41 M_3_ rice plants mutagenized by fast neutron irradiation. Since the genome size of rice is about three times that of Arabidopsis, the MF per site is comparable to those in Arabidopsis. During the reviewing process of this paper, another study was published on the whole-genome mutational analysis in M_3_ generation of Arabidopsis following carbon ion irradiation with the mean LET of 50 keV/μm^19^. They reported that the estimated mutation frequency in M_1_ generation was 3.4 × 10^−7^/bp and the number of variations per line in M_3_ generation ranged from 20 to 62. These values are comparable to the data obtained here.

In this study, the proportions of the types of mutations in dry-seed irradiation were 38–43% SBS, 43–48% deletion, 7% insertion, and 7% complex type (Table 1). In Arabidopsis subjected to fast neutron irradiation, the proportions of SBS, deletions, and insertions were 59%, 36%, and 5% (out of a total of 108 mutations), respectively^9^. In rice, the proportions of SBS, deletions, and insertions were 53%, 36%, and 6% (out of total 2,418 mutations), respectively^10^. In Arabidopsis subjected to carbon ion irradiation (50 keV/μm), 320 SBS and 124 InDels were detected in 11 M_3_ plants^19^. The proportion of SBS (72%) was higher than the above values. Although the irradiation and analytical methods might affect these results, they reveal a general trend that fewer SBS and more deletion mutations are induced as the LET of ionizing radiation increases.

The G to A transition was the main SBS in both dry-seed irradiation and seedling irradiation (Fig. 3). Interestingly, the A to T transversion was frequently detected in dry-seed irradiation but rarely detected in seedling irradiation. Du *et al*.^19^ also reported that the G to A transition was the major SBS in dry-seed irradiation with carbon ions in Arabidopsis. They also observed the relatively higher proportion of A to T transversion. The G to A transition was also the major SBS in Arabidopsis dry seeds subjected to fast neutron irradiation, followed by the G to T transversion, and the A to T transversion was much less common^9^. In fast neutron irradiation of rice seeds, the G to A transition was the major SBS, while G to T and A to T transversions were much less common^10^. Thus, the G to A transition is often a main mutation, but the proportions of the other types of SBS are inconsistent. We think oxidative damage to guanine would not be a major cause of the high frequency of G to T transversions observed in dry-seed irradiation with fast neutron in Arabidopsis. This is because we did not observe an increased frequency of G to T transversions in seedling irradiation (Fig. 3), in which high concentrations of hydroxyl radicals and other reactive oxygen species and increased incorporation of damaged bases were anticipated. Yoshihara *et al*.^20,21^ also described that oxidative damage to guanine had little effect on mutagenesis in Arabidopsis. They performed a plasmid rescue experiment to compare the radiation-induced mutations in dry seeds and seedlings. The frequency of G to T and A to C transversions, which could be caused by oxidized guanine, were less common in both materials. The mechanism underlying the higher frequency of A to T transversions in dry-seed irradiation than in seedling irradiation (Fig. 3) also remains unclear. The chemical mutagen diepoxybutane frequently induces A to T transversions^22^, but it is unclear whether the physiological status of plant tissue affects the types and yield of base damage induced by carbon-ion irradiation. In this study, the Ti/Tv ratio was in the range of 0.67 to 0.88 and did not differ significantly among the experimental groups (Fig. 3). Du *et al*.^19^ reported that the Ti/Tv ratio was 0.99 in dry-seed irradiation with carbon ions in Arabidopsis. The Ti/Tv ratios reported for Arabidopsis and rice subjected to fast-neutron irradiation were 0.86 and 1.4, respectively^9,10^. These values were markedly lower than that of spontaneous mutation (2.4)^18^. The G to A transition, which is mostly caused by the deamination of methylated cytosines, is a predominant spontaneous mutation, while an increased frequency of transversion mutations is commonly observed in carbon ion and fast neutron irradiation.

Most of the deletions detected in this study were smaller than 50 bp (Fig. 4). The distribution of deletion size was similar to that observed after fast neutron irradiation of Arabidopsis^9^. Similarly, 73% of deletions were reported to be smaller than 10 bp in rice^10^. Deletions induced by carbon ions with the LET of 50 keV/μm was 21 bp or smaller in that single base deletion was predominant (66%, 68/103)^19^. Large deletions and complex SVs are often observed after argon-ion irradiation, which has a higher LET (290 keV/μm) than that of the carbon ion irradiation used here (107 keV/μm)^11^. This corresponds with the current understanding that large deletions and SVs increase as the LET of ion beams increases^2,23^. These results suggest that the carbon ions with LET ranging from 50 to 100 keV/μm and fast neutrons often induce relatively small deletions, although we cannot exclude the possibility that large deletions are detected less efficiently with the current analytical methods.

We observed that single base insertions and deletions were often in a homopolymeric sequence, while ≥2 bp InDel mutations were often associated with polynucleotide repeats or microhomologous sequences (Table 2). It is thought that InDels induced by ionizing radiation are mostly caused by misalignment during DNA replication or non-homologous end-joining (NHEJ), an error-prone repair mechanism^24^. An Arabidopsis mutant with a disrupted DNA ligase IV, a key component of the NHEJ pathway, was shown to be 4.5 times more sensitive than wild type to carbon-ion irradiation^5^. This supports the view that the NHEJ is a major repair pathway for DNA damage induced by carbon ion irradiation. Furthermore, several other studies have suggested that the NHEJ pathway typically utilizes short homologies for rejoining. Shikazono *et al*.^3^ described that short (1–5 bp) homologous sequences were found in 12 out of 19 rejoined sites of SV induced by carbon-ion irradiation to Arabidopsis dry seeds. They also mentioned that four out of five InDels with the sizes of 1 and 2 bp were found within a run of repeated sequences; hence, at some of them arose from errors during DNA synthesis. Belfield *et al*.^9^ described that among the 14 single base deletion mutations induced by fast-neutron irradiation to Arabidopsis dry seeds, five were in homopolymeric sequences and four were in polynucleotide repeats. In the study of Belfield *et al*.^9^, among the 22 of InDels larger than 2 bp, 11 involved microhomologous sequences at the junction, while five were associated with homopolymeric sequences. Hirano *et al*.^11^ re-sequenced three Arabidopsis mutant lines obtained by argon-ion irradiation and reported that 62% of the SV junctions contained microhomologous sequences 1–7 bp long. Yoshihara *et al*.^20^ identified 1–7-bp microhomologous sequences in 8 out of 17 deletions detected by a plasmid rescue experiment on Arabidopsis dry seeds irradiated with carbon ions. These results do not contradict the characteristics of the sequences observed in this study. Du *et al*.^19^ also reported a similar characteristics in Arabidopsis irradiated with carbon ions. Furthermore, we detected differences in the characteristics of sequences between single-base and ≥2 bp InDels. In the case of dry-seed irradiation, the ratios of homopolymeric sequences associated with 2-bp deletions (3/17) and 3-bp deletions (1/18) were markedly lower than that of homopolymeric sequences associated with single base deletions (22/49) (Tables 2, S5). In the case of seedling irradiation, half of the −1 deletions (8/16) and both 2-bp deletions occurred in homopolymeric sequences, but none of the 17 3–9-bp deletions were in homopolymeric sequences. These observations suggest that different mechanisms are involved in the generation of single base deletions and deletions larger than 2–3 bp. We also observed an interesting trend that microhomology was less commonly associated with deletions of ≥50 bp (Table 2). This suggested that the repair process may differ depending on the length of the deletion. Although the underlying mechanism remains unclear, further progress is expected by whole-genome analyses of repair-deficient strains and after different types of mutagenesis treatments.

Isolation and analysis of mutants with a visible phenotype was the most straight-forward method to analyse the characteristics of mutations, until the emergence of next generation sequencing technology. Shikazono *et al*.^3^ examined 104,088 Arabidopsis M_2_ plants derived from 26,200 dry seeds mutagenized with 150 Gy of carbon-ion irradiation (107 keV/μm), and obtained 12 mutants at the *TT* locus or the *GL* locus. All of them were homozygous for the mutant allele, resulting in altered seed colour or a trichome-less phenotype. The MF in the M_2_ generation was calculated to be 7.0 × 10^−5^/locus (88 mutants/(104,088 plants × 12 loci)). This suggested that 1.9 genes per plant had a homozygous loss-of-function mutation among the 27,206 protein coding genes in the TAIR10 genome. In this study, an average of 7.0 and 13.3 protein coding genes were predicted to be affected by a homozygous mutation after 125-Gy and 175-Gy dry seed irradiation, respectively (Table 3). The MF calculated from the phenotype-based analysis does not include cases where more than two genes are affected by a single mutation event. If these cases are excluded, the number of affected genes in this study would be 1.8 and 3.5, respectively. This suggests that a comparable number or slightly more gene mutations are detected by whole genome-sequencing analysis than by a phenotype-based analysis.

This study demonstrated for the first time that the physiological status of plant tissue greatly affects the frequency and types of mutations induced by high-LET radiation at the molecular level. These findings are highly relevant for developing more efficient mutation breeding strategies and also for understanding the molecular mechanism of mutagenesis by ionizing radiation.

## Materials and Methods

### Plant growth conditions, ion-beam irradiation, survival rate, and fertility

We used seeds of *Arabidopsis thaliana* (Columbia (Col-0) accession) obtained from a single plant to exclude the background mutations that exist in our laboratory strain. Plants were grown in pots (5 cm W × 5 cm D × 5 cm H) or plug trays (200 cells/tray, Takii & Co., Ltd., Kyoto, Japan) filled with a 1:1 mixture of culture soil (TM-2, Takii) and vermiculite (Medium size, Vern-piece, Hakugen Co., Ltd., Tokyo, Japan), unless otherwise indicated. Dry seeds and 7-day-old seedlings grown in 6-cm diameter plastic dishes filled with TM-2 culture soil were irradiated with 17.3 MeV/u carbon ions (surface LET, 107 keV/μm; calculated range in water, 1.1 mm) as previously described^5^. Irradiated seedlings were transplanted into plug trays and the survival rate was determined 3 weeks after irradiation. Irradiated dry seeds were sown and the survival rate was determined 1 month after sowing. Three replications of 25 seeds or seedlings were used for each dose. Survival curves were drawn and the shoulder dose (*Dq*) was calculated as previously described^5^. Seed fertility was determined as the ratio of the number of fertilized ovules to the total number of ovules on one side of the septum for each silique. These analyses were conducted using the 3rd silique from the base, or the 4th or 5th silique if the 3rd or both the 3rd and 4th siliques were sterile. The plants were judged as sterile if the 3rd, 4th, and 5th siliques were sterile. More than 50 plants were examined for each dose. Seeds of the following generation (M_2_ lines) were obtained from individual plants by self-pollination.

### Whole genome re-sequencing

Six M_2_ lines were randomly chosen for each of the four experimental groups (Dry seed_125 Gy, Dry seed_175 Gy, Seedling_20 Gy and Seedling_30 Gy). To ensure random selection of plants regardless of the phenotype, five seeds from each M_2_ line were sown in pots and numbered 1 to 5. If the number 1 seed did not germinate or grow well enough to obtain sufficient DNA, another plant was selected in numerical order. Genomic DNA was extracted from rosette leaves using the MagExtractor Plant Genome DNA extraction kit according to the manufacturer’s instruction (Toyobo Co. Ltd., Tokyo, Japan). Genomic DNA was fragmented using NEBNext dsDNA fragmentase (New England Biolabs Japan Inc. Tokyo, Japan), and a sequencing library was prepared using the KAPA HTP Library Preparation Kit and SeqCap Adapter Kit (Nippon Genetics Co. Ltd, Tokyo, Japan). The length, concentration, and purity of DNA fragments were assessed using an Agilent 2100 Bioanalyzer (Agilent Technologies Japan, Ltd., Tokyo, Japan). Paired-end reads (150 bp) were obtained using an Illumina NextSeq500 system by the DNA Chip Research Inc., Tokyo, Japan. The raw paired-end reads were cleaned by removing low quality reads and Illumina adaptor sequences using Trimmomatic (version 0.33, http://www.usadellab.org/cms/?page=trimmomatic). The cleaned data were mapped to the Arabidopsis reference genome (TAIR10.27, https://www.arabidopsis.org/) using BWA (version 0.7.5, http://bio-bwa.sourceforge.net/), SAMtools (version 1.3.1, http://samtools.sourceforge.net/), and Picard-tools (version 1.119, https://broadinstitute.github.io/picard/). The candidate mutations were identified using GATK HaplotypeCaller (version 3.4, https://software.broadinstitute.org/gatk/), Pindel (version 0.2.4, http://gmt.genome.wustl.edu/packages/pindel/user-manual.html), and BreakDancer (version 1.4.5, http://breakdancer.sourceforge.net/) algorithms. The identified mutations were annotated using SnpEff (version 4.1, http://snpeff.sourceforge.net/). For the GATK analyses, the candidate mutation sites with at least three mutant reads were further considered. The candidate mutations that were found in more than two independent samples were excluded as background mutations. The candidate mutation site was defined as heterozygous if the allele frequency (proportion of mutant reads at a site, AF) was >25% to <80%, and if the AFs for all the other 23 samples were <5%. The candidate mutation site was defined as homozygous if the AF ≥80%, and if the AFs for all the other 23 samples were <5%. For the Pindel and BreakDancer analyses, the candidate mutation sites were sorted by the position in the genome and those unique to a single sample were further considered. All candidate mutations were confirmed by the Integrative Genomics Viewer (IGV) (Version 2.3.40, http://software.broadinstitute.org/software/igv/). The sequence of each read was also confirmed as necessary.

### Verification of identified mutation

Some of the identified mutation sites were verified by Sanger sequencing. We designed 41 primer pairs to amplify mutation sites, and 37 of them successfully amplified a single band of the expected length from wild-type genomic DNA. The sequences of the mutation sites were verified using the 37 primer pairs (Table S2) with the remaining M_2_ plant DNA, which was used for whole genome re-sequencing. The PCRs were performed in a GeneAmp PCR System 9700 (Applied Biosystems, Tokyo, Japan) using 20 ng genomic DNA, 0.1 μM each of forward and reverse primer, 150 μM each dNTP, 0.5 unit Ex Taq polymerase (Takara Bio Inc., Shiga, Japan) and 1× Ex Taq buffer in a 20-μl reaction volume. The PCR conditions were as follows: initial denaturation at 94 °C for 5 min followed by 40 cycles of 94 °C for 30 s, 58 °C for 30 s, and 72 °C for 1 min, and final extension at 72 °C for 2 min. Each amplified fragment was purified using a MinElute PCR purification Kit (Qiagen K. K., Tokyo, Japan), and sequenced using the Big Dye Terminator v3.1 Cycle Sequencing Kit (Thermo Fisher Scientific K. K., Yokohama, Japan) and a 3500 Genetic Analyzer (Applied Biosystems) according to the manufacturers’ instructions.

## Electronic supplementary material

Supplementary Information
