# Supplementary material for: Physiological status of plant tissue affects the frequency and types of mutations induced by carbon-ion irradiation in Arabidopsis
Source: Sci Rep. 2018 Jan 23;8:1394. doi: 10.1038/s41598-018-19278-1 (PMC5780457; doi:10.1038/s41598-018-19278-1)
Supplement: Supplementary file 1 — Supplementary Information [file 41598_2018_19278_MOESM1_ESM.pdf]

Physiological status of plant tissue affects the frequency and types of mutations induced by carbon-ion irradiation in Arabidopsis

–Supplementary Information–

Yoshihiro Hase, Katsuya, Satoh, Satoshi Kitamura, Yutaka Oono

Takasaki Advanced Radiation Research Institute, National Institutes for Quantum and Radiological Science and Technology (QST), 1233 Watanuki, Takasaki, Gunma 370-1292, Japan

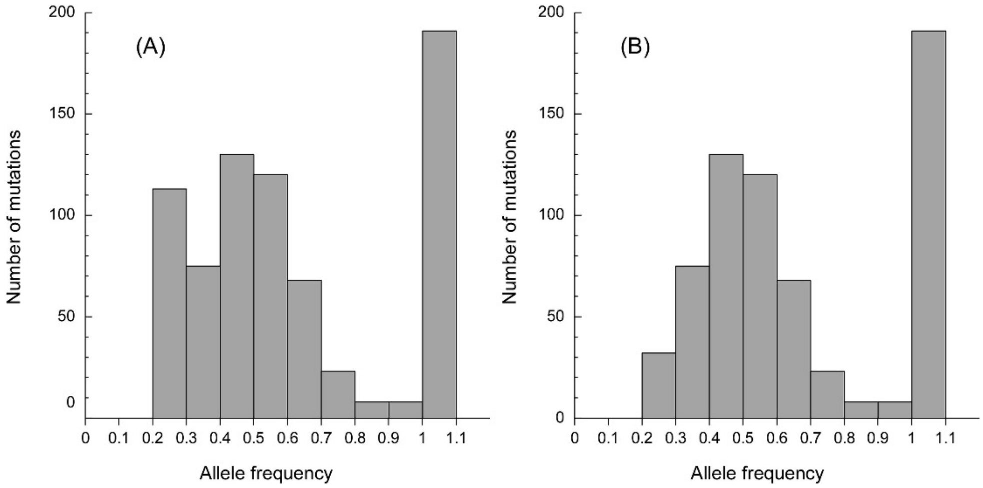

Figure S1. Distribution of allele frequencies for candidate mutations detected by GATK HaplotypeCaller algorithm. All candidate mutations with allele frequencies >0.20 (A) and >0.25 (B) are shown.

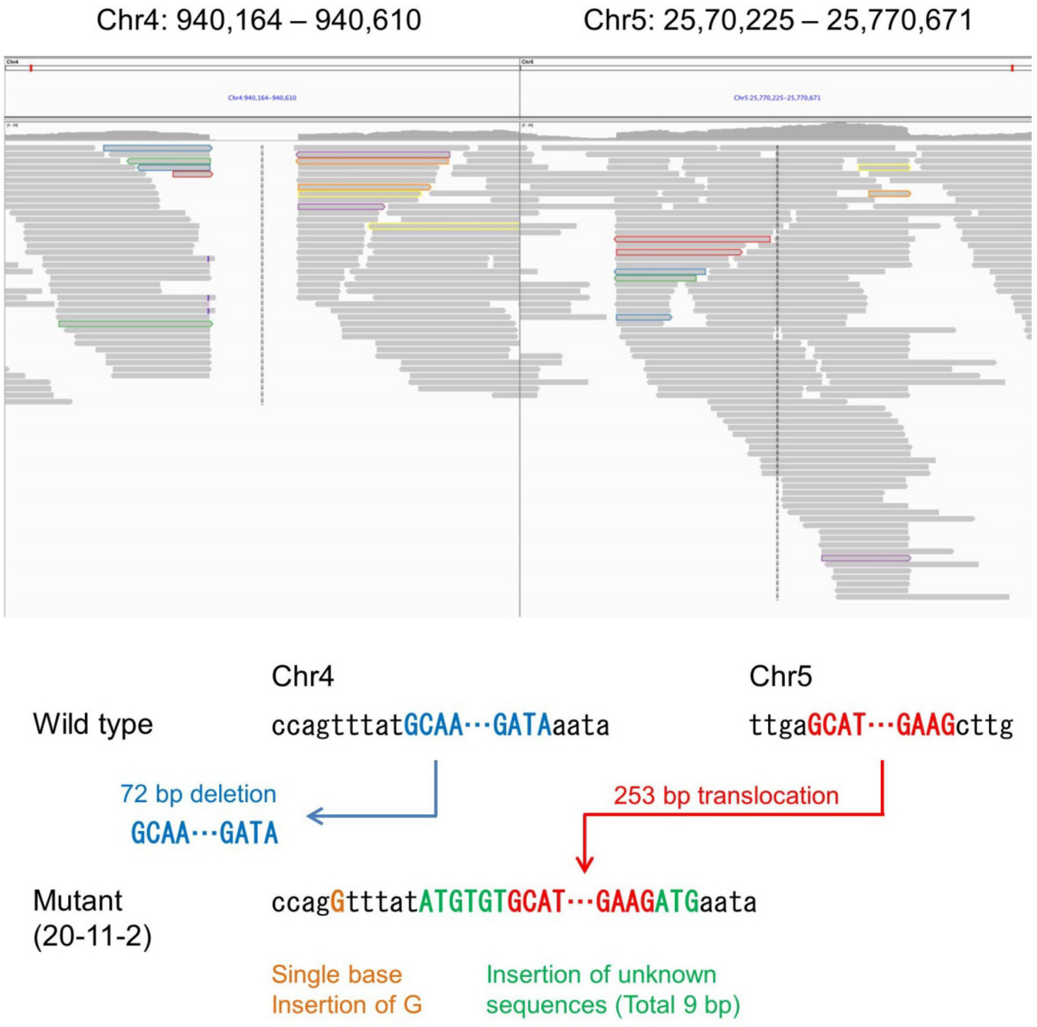

Figure S2. Structural variation identified in sample 20-11-2. (Upper) Captured screen image of IGV at two junction points of structural variation identified in sample 20-11-2. Several paired sequence reads are shown in the same colour, indicating inter-chromosomal connection between chromosomes 4 and 5. Increased depth in 253-bp region in chromosome 5 suggests duplication of this region in the genome, resulting from translocation from chromosome 5 to 4. (Lower) Sequence alteration confirmed by Sanger sequencing. Confirmed alteration was identical to that predicted from image of IGV and sequences of paired reads. The primer sequences used here were 5’-CTCATGGGCAGAAAAGTCGT-3’ and 5’-GCTTGTTTTACTTCGGTTTCG-3’.

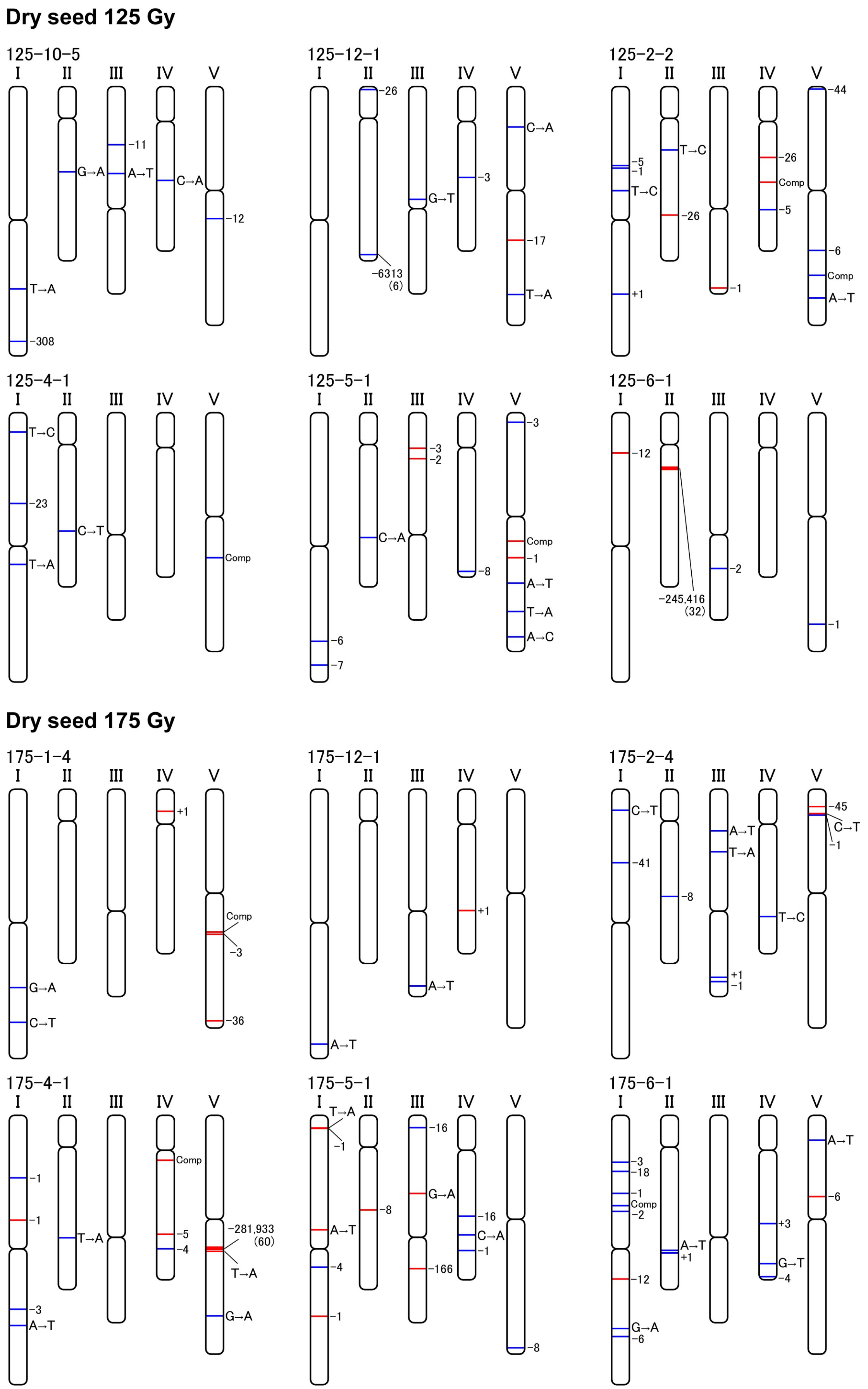

Figure S3. Distribution of mutation events that resulted in non-synonymous amino acid changes in protein-coding genes across chromosomes. Red and blue colour represent homozygous and heterozygous mutations, respectively. Transposable elements and pseudogenes are not included. Number of affected genes is shown in parenthesis if more than two genes are affected by a single mutation event.

Seedling 20 Gy

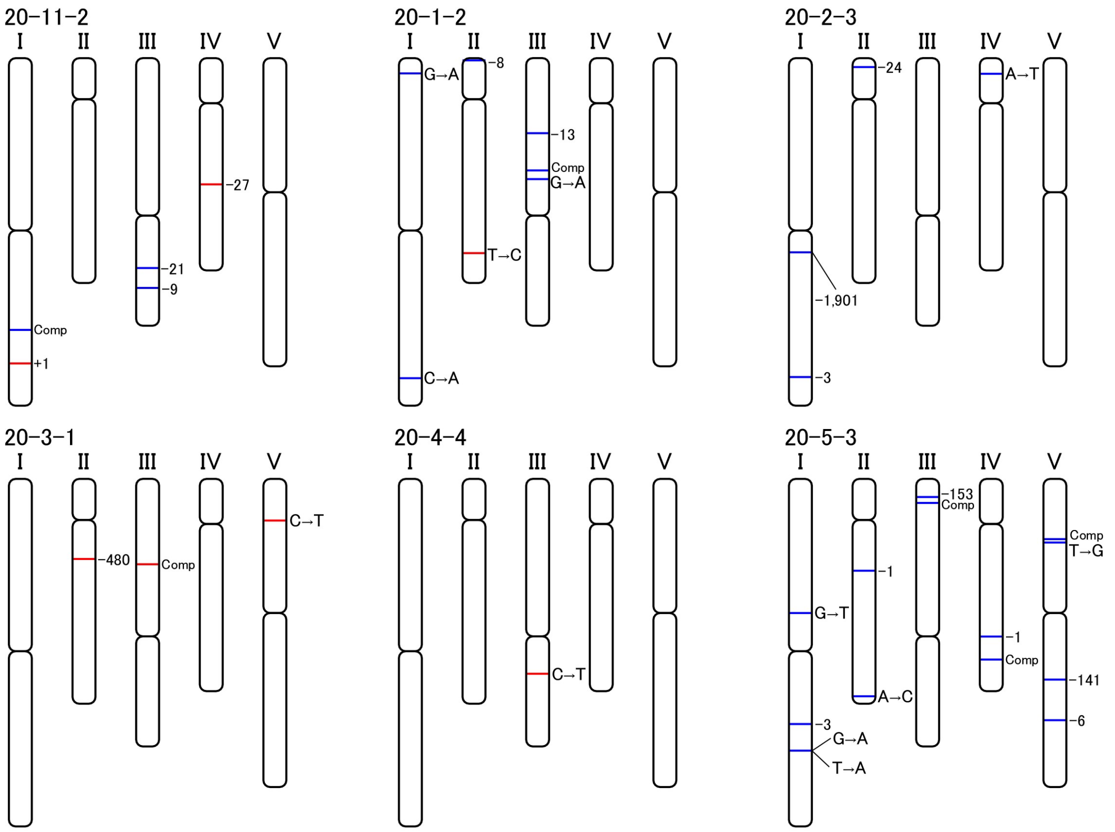

Seedling 30 Gy

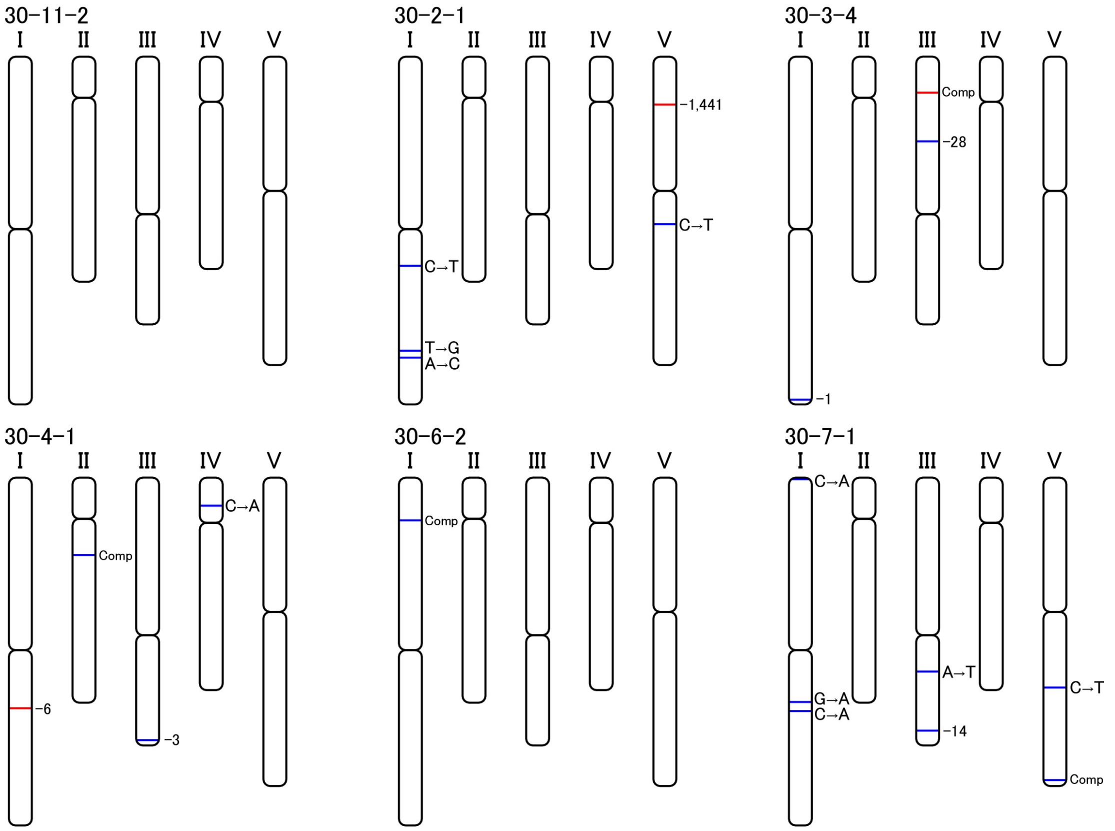

Figure S3. (Continued.)

Table S1. Summary of mapping results.

Sample	Total mapped base (Mb)	Mean depth of coverage	Coverage above 10x (%)
Dry seed 125 Gy			
125-10-5	4,533	37.9	99.4
125-12-1	3,962	33.2	98.4
125-2-2	3,258	27.3	93.1
125-4-1	4,683	39.2	99.4
125-5-1	4,143	34.7	99.0
125-6-1	4,029	33.7	98.9
Dry seed 175 Gy			
175-12-1	3,799	31.8	98.0
175-1-4	4,179	35.0	98.6
175-2-4	3,621	30.3	97.4
175-4-1	3,891	32.6	98.5
175-5-1	4,571	38.3	99.5
175-6-1	4,326	36.2	98.8
Seedling 20 Gy			
20-11-2	4,595	38.5	99.5
20-1-2	3,214	26.9	96.7
20-2-3	3,776	31.6	98.6
20-3-1	3,957	33.1	99.0
20-4-4	4,015	33.6	98.1
20-5-3	3,529	29.5	97.7
Seedling 30 Gy			
30-11-2	3,396	28.4	95.9
30-2-1	4,460	37.3	99.4
30-3-4	4,475	37.5	99.5
30-4-1	3,654	30.6	98.1
30-6-2	3,542	29.7	98.0
30-7-1	3,570	29.9	97.3
Mean	3,966	33.2	98.2
SD	443	3.7	1.4

Table S2. Results of verification of identified mutations.

Sample	Chr	Position	Type of Mutation	Detail	Zygosity	Result	Forward and reverse primer (5'→3')	Allele Frequency*
125-10-5	1	3,622,144	SBS	T→A	Hetero	True	F: AACAGCATAGGGTTGGTTGG<br>R: TTTTGCATTCCAGACTGTCG	0.55 (12/22)
125-10-5	1	20,029,686	SBS	A→T	Hetero	True	F: TGCGAGTAGAATGCACTTGG<br>R: AATTGCAGGCCACTGAAGAT	0.58 (21/36)
125-10-5	1	23,593,771	SBS	T→A	Hetero	True	F: AGGAGCATTCTGCCAAAGAA<br>R: CCAGTCGAAGGAAGGATCTG	0.71 (25/35)
125-10-5	2	7,178,100	SBS	T→G	Homo	True	F: CGACCCAAACTCGTCATACC<br>R: GCATTTCCTTATCTAATATTCCTGATG	1.00 (40/40)
125-10-5	2	9,957,746	SBS	G→A	Hetero	True	F: ACCACACCCACGAAGTTCTC<br>R: ACCTTTGGCGATGATGAAAC	0.37 (13/35)
125-10-5	2	17,637,661	SBS	A→T	Homo	True	F: GGCGTTTTCTCCATCTTCTG<br>R: AATGGGCTAAAAGGGACACC	1.00 (19/19)
125-10-5	3	6,715,992	SBS	C→A	Hetero	True	F: GGAACGAGAGCTTATCTGGGTA<br>R: GCGACTTTCTCCCACAAAAC	0.32 (9/28)
125-10-5	3	10,132,131	SBS	A→T	Hetero	True	F: AAGCAGCTGGACCAAGGTAA<br>R: ATTGAGCCGTGGTTGAAGAG	0.55 (16/29)
125-10-5	4	10,949,870	SBS	C→A	Hetero	True	F: CAAGATTCGTGTCGGACAAA<br>R: TGGAATGTTCCATGAATCAAGA	0.42 (8/19)
125-10-5	4	11,953,215	SBS	A→T	Hetero	True	F: AAAAACGTTGCAAAAACAACA<br>R: CGGTTGGTTCGATCAGTTTA	0.51 (18/35)
**125-10-5**	**2**	**13,338,270**	**-1**	**A**	**Hetero**	**True**	**F: CGACTACGAGGGACACTTGC**<br>**R: TTTTCGAGGCTCATTCGTCT**	**0.23 (5/22)**
20-11-2	5	556,406	-1	G	Hetero	True	F: AACGTAAGATGGCGTGGTTC<br>R: ACAAACCCTAGCCGCCTTAT	0.59 (23/39)
20-11-2	5	10,291,632	-1	A	Hetero	True	F: AATGGGGATTCATTGGGTTT<br>R: CGATTTTGCCCTCATTCTTC	0.39 (9/23)
175-1-4	1	8,176,912	-1	G	Hetero	True	F: TTTGGGAATTTTTGTGTGGA<br>R: ATATGCCAACGCCATTCTTC	0.40 (8/20)
175-1-4	2	10,388,368	-1	A	Homo	True	F: GGATGAGTTTGCGTGTGTGT<br>R: GACGTCGGAAAAAGTTTGGA	1.00 (28/28)
175-1-4	5	84,346	-1	A	Hetero	True	F: GGCAAGAATGTGCGTTTGAT<br>R: GTCGGATCAATGGTTGGATT	0.44 (12/27)
175-1-4	5	7,195,682	-1	T	Homo	True	F: AACCTTTCCCCAATCAAAGG<br>R: AATTCCTTTCTCCCGTCCAC	1.00 (23/23)

**175-1-4**	**5**	**20,942,238**	**-1**	**A**	**Hetero**	**False**	**F: TGCCTGAATTTTGAAATGCTC**<br>**R: CGTGTGTATACCAATAATCGTTTC**	**0.25 (4/16)**
125-10-5	1	25,115,764	Del_≥2 bp	-2 bp	Hetero	True	F: ATCAGCCCACCAAGAAATTG<br>R: GCTGCCACTGCATACTCAGA	0.38 (8/21)
125-10-5	1	29,787,264	Del_≥2 bp	-308 bp	Hetero	True	F: GGGTTCCAACCAACAAGATG<br>R: CAGGCTACTAAAAATAATGCCAAT	
125-10-5	2	6,248,477	Del_≥2 bp	-22 bp	Hetero	True **	F: TTCCCAAAAGGTGTGACTTT<br>R: GCGCCTATCATGATGTGATT	0.40 (14/35)
125-10-5	3	6,821,869	Del_≥2 bp	-11 bp	Hetero	True **	F: GAAGGATGCGAAGCAAAGTC<br>R: AGGGCTGGATGAGCCTCTA	0.39 (12/31)
125-10-5	3	16,755,265	Del_≥2 bp	-2 bp	Hetero	True	F: TGTTGGTCGGTAAAAGACGA<br>R: AGAGGGCCTTGCAGAAGTTT	0.47 (7/15)
125-10-5	4	11,649,411	Del_≥2 bp	-144 bp	Hetero	True **	F: GGTCGATTTGCAATTACCTGA<br>R: CACATGGATCAAGATTCATTCAATA	
125-10-5	5	15,422,503	Del_≥2 bp	-12 bp	Hetero	True **	F: ATCGCTGCCTCAGGTCTAAA<br>R: TTGTTCATACCACCAGCACAA	0.29 (6/21)
20-11-2	1	26,986,773	+1	T	Homo	True	F: GAAGCGTTGTTTGGGATCTG<br>R: GAACCATGCGTCTTCACTCA	1.00 (25/25)
30-11-2	1	18,032,053	+1	A	Homo	True	F: CGAACCCTTCAATCATTGGT<br>R: TGAGAGGCTTTCCTCCTTGA	1.00 (18/18)
175-1-4	4	2,556,689	+1	TCG→AACT	Homo	True	F: AGAGTCAAAGCCGAAAAGCA<br>R: GACGGAATCCTCTCCATCAA	1.00 (32/32)
**20-11-2**	**5**	**638,204**	**Ins_≥2bp**	**+2 bp**	**Hetero**	**False**	**F: CAAACCAGACCAGACAACGA**<br>**R: CGCTGCAACTGATGATAACG**	**0.22 (4/18)**
125-12-1	1	20,607,208	Ins_≥2bp	+27 bp	Homo	True	F: AGATTCTCCGGCGATACCTC<br>R: GTCCGATTCCTGATTCTCCA	1.00 (14/14)
30-11-2	1	8,024,381	Ins_≥2bp	+47 bp	Homo	True	F: ATCCAACGTGTTTCGTAGCC<br>R: TTCGAAGACAAATCCATCAAAA	1.00 (10/10)
175-1-4	2	17,134,939	Ins_≥2bp	+5 bp	Hetero	True	F: TCAGAGGATGGAGTGGGTTC<br>R: CTGGGAAGTGCTTCAACGTC	0.57 (12/21)
175-1-4	3	1,064,359	Ins_≥2bp	+2 bp	Hetero	True	F: TTGTTTTGAATGCGATGCTC<br>R: TTTGAGGGAAGAAGAAAATCACTT	0.70 (16/23)
125-10-5	2	5,641,286	Complex	CC→AT	Hetero	True	F: GCCCCTAAGCGTGTATTTGT<br>R: GCCTGAGGAAGGATATCTGATG	0.48 (11/23)
20-11-2	1	20,029,121	Complex	GCA→AGT	Hetero	True	F: TCCATTGACCCAAAAACTCC<br>R: TGGAAAGACAACAACCATGAA	0.43 (12/28)
20-11-2	1	24,023,841	Complex	See Table S6 for detail	Hetero	True **	F: GGGTCAGCTTCTGAGGTCAT<br>R: TCTGCAATTCCGTATCATGC	

20-11-2	3	2,999,203	Complex	See Table S6 for detail	Hetero	True **	F: AGCTCCGTTGACCAAAAAGA<br>R: TTTCACGTAATATCAAACATCCAA	0.34 (10/29)

Candidate mutations with allele frequencies of ≤0.25, which were excluded from this study, are shown in bold.

* Allele frequencies are not shown for those detected by Pindel and BreakDancer algorithms. Numbers in parentheses represent number of mutant alleles/number of total alleles at the site.

** Only junction sequences (~5 bp) were confirmed.

Table S3. Predicted structural variations caused by irradiation treatments.

Structural variations identified in dry-seed irradiation.

Sample	Deduced structural variation
125-4-1	Intra-chromosomal inversion (chr4, ca. 312 kbp)
125-6-1	Intra-chromosomal inversion (chr2, ca. 521 kbp)
125-6-1	Intra-chromosomal inversion (chr4, ca. 207 kbp)
175-2-4	Intra-chromosomal inversion (chr1, ca. 11 kbp)
175-2-4	Intra-chromosomal inversion (chr3, ca. 382 kbp)
175-5-1	Intra-chromosomal translocation (chr3, ca. 4168 kbp and 179 kbp)
175-4-1	Inter-chromosomal translocation (chr2 and 5) *
175-4-1	Inter-chromosomal translocation (chr3 and 5) *

* Failed to predict overall structure.

Structural variations identified in seedling irradiation.

Sample	Deduced structural variation
20-11-2	Inter-chromosomal translocation (chr5 to 4, ca. 253 bp)**
20-1-2	Reciprocal translocation (chr1 and 2)
20-1-2	Inter-chromosomal translocation (chr3 to 4) *
20-1-2	Intra-chromosomal inversion (chr5, ca. 14 kbp)
20-2-3	Intra-chromosomal inversion (chr2) *
20-2-3	Intra-chromosomal inversion (chr5) *
20-4-4	Intra-chromosomal inversion (chr2, ca. 431 kbp with 26-kbp deletion)
30-11-2	Intra-chromosomal translocation (chr1, ca. 1 kbp) and inversion (chr1, ca. 38 kbp)
30-11-2	Intra-chromosomal translocation (chr5, ca. 705 kbp) and inversion (chr5, ca. 573 kbp)
30-2-1	Inter-chromosomal translocation (chr3 to 1, ca. 500 bp)
30-4-1	Intra-chromosomal translocation (chr4, ca. 255 kbp)

* Failed to predict overall structure.

** Details are shown in Supplemental Figure S2.

Table S4. Zygosity of identified mutations.

Dry-seed 125 Gy							
Sample	125-10-5	125-12-1	125-2-2	125-4-1	125-5-1	125-6-1	Total
Homo	2	17	9	6	14	15	63 (10.5 ± 2.4)
Hetero	18	19	30	24	20	16	127
Total	20	36	39	30	34	31	190 (31.7 ± 2.7)
Hetero / homo ratio	9.0	1.1	3.3	4.0	1.4	1.1	(3.3 ± 1.2)
Dry-seed 175 Gy							
Sample	175-1-4	175-12-1	175-2-4	175-4-1	175-5-1	175-6-1	Total
Homo	19	6	8	15	27	8	83 (13.8 ± 3.3)
Hetero	12	21	39	23	23	27	145
Total	31	27	47	38	50	35	228 (38.0 ± 3.7)
Hetero / homo ratio	0.6	3.5	4.9	1.5	0.9	3.4	(2.5 ± 0.7)
Seedling 20 Gy							
Sample	20-11-2	20-1-2	20-2-3	20-3-1	20-4-4	20-5-3	Total
Homo	7	8	4	9	4	3	35 (5.8 ± 1.0)
Hetero	15	19	17	9	7	30	97
Total	22	27	21	18	11	33	132 (22.0 ± 3.1)
Hetero / homo ratio	2.1	2.4	4.3	1.0	1.8	10.0	(3.6 ± 1.4)
Seedling 30 Gy							
Sample	30-11-2	30-2-1	30-3-4	30-4-1	30-6-2	30-7-1	Total
Homo	5	7	4	3	1	0	20 (3.3 ± 1.1)
Hetero	15	16	8	14	11	33	97
Total	20	23	12	17	12	33	117 (19.5 ± 3.2)
Hetero / homo ratio	3.0	2.3	2.0	4.7	11.0	- *	(4.6 ± 1.5**)

Structural variations are not included. Numbers in parentheses represent mean ± standard error of 6 samples in each group.

* Ratio could not be calculated, because all identified mutations were heterozygous.

** Data from sample 30-7-1 were not included in calculation.

Table S5. Details of insertion and deletion mutations.

Single-base deletions identified in dry-seed irradiation.

Sample	Chr	Position	Type of mutation	Sequence
125-12-1	1	297,283	-1	ccca**T**aaga
125-12-1	1	922,788	-1	tgat**A**aaat
125-12-1	1	4,915,941	-1	tctc**A**agtc
125-12-1	1	7,320,957	-1	tttc**A**agtt
125-12-1	2	11,837,433	-1	gccc**A**aaaa
125-12-1	3	16,902,286	-1	caga**T**tttt
125-12-1	3	19,919,338	-1	ctat**A**atcg
125-2-2	3	18,875,489	-1	gaag**A**aaaa
125-2-2	3	23,408,189	-1	aaca**T**tatc
125-2-2	4	12,094,794	-1	aata**T**tttt
125-2-2	4	13,326,642	-1	ataa**T**tatg
125-2-2	5	19,338,359	-1	acca**C**aaaa
125-4-1	3	9,542,017	-1	tttc**T**tgtt
125-4-1	5	23,187,571	-1	aata**T**ttta
125-5-1	1	22,356,811	-1	gggg**A**aaaa
125-5-1	1	28,796,972	-1	acta**G**ggag
125-5-1	5	16,600,638	-1	tgat**G**cctc
125-6-1	2	5,459,889	-1	aaat**A**aaaa
125-6-1	3	17,202,547	-1	atat**C**tttt
125-6-1	5	22,930,227	-1	ccca**T**tttt
125-6-1	5	23,294,994	-1	tgag**T**tttt
125-6-1	5	24,196,261	-1	tacc**G**agcg
175-12-1	2	16,477,858	-1	tttc**T**tcct
175-12-1	3	17,552,485	-1	gtca**T**cttc
175-12-1	3	21,676,609	-1	cgat**G**atgc
175-12-1	4	4,159,520	-1	tgga**G**gggg
175-1-4	1	8,176,912	-1	acat**G**tttt
175-1-4	2	10,388,368	-1	gtat**A**aacg
175-1-4	5	84,346	-1	aaag**A**aaaa
175-1-4	5	7,195,682	-1	tatc**T**tttg
175-2-4	1	12,931,508	-1	atta**T**taat
175-2-4	2	10,972,839	-1	gata**T**tttt
175-2-4	2	13,056,328	-1	ctcc**T**tttt
175-2-4	3	19,760,337	-1	atga**T**gtgc
175-2-4	3	22,154,527	-1	gaaa**T**caag
175-2-4	5	2,906,800	-1	cacc**G**acgg
175-4-1	1	7,190,092	-1	agtt**G**gtaa
175-4-1	1	11,943,800	-1	atct**C**ccaa
175-4-1	1	21,463,635	-1	ttaa**C**caaa
175-4-1	2	14,488,918	-1	tttt**C**ccca
175-4-1	5	3,490,938	-1	caaa**T**tttt

175-5-1	1	1,483,693	-1	ccgc**T**agta
175-5-1	1	1,707,003	-1	atac**T**tttc
175-5-1	1	22,959,254	-1	aact**G**actt
175-5-1	2	13,089,815	-1	cata**G**ttca
175-5-1	3	11,744,819	-1	gtgc**Aa**taa
175-5-1	4	4,876,808	-1	agtc**A**tttg
175-5-1	4	15,554,563	-1	tgct**A**aagt
175-6-1	1	8,881,852	-1	aagt**G**agaa

Deleted bases are shown in bold. Homopolymeric sequences are underlined. Polynucleotide repeats are boxed. Two of -1 mutations that are expected to be induced by 2-bp deletion followed by 1-bp insertion are not included.

Single base deletion identified in seedling irradiation.

Sample	Chr	Position	Type of mutation	Sequence
20-11-2	5	556,406	-1	agaa**G**acga
20-11-2	5	10,291,632	-1	aaat**A**aaaa
20-2-3	4	2,339,717	-1	tcat**A**aaca
20-2-3	4	12,199,215	-1	aaga**G**gctc
20-4-4	3	18,249,789	-1	cagt**C**acac
20-5-3	2	8,178,652	-1	tgga**T**actt
20-5-3	4	16,096,073	-1	acta**G**ggct
20-5-3	4	17,044,669	-1	atga**T**tttc
30-11-2	1	3,488,232	-1	tatc**T**tttt
30-11-2	4	4,974,796	-1	gatc**T**tatg
30-2-1	5	15,129,107	-1	cgtg**A**aatg
30-3-4	1	30,318,438	-1	aggg**T**taaa
30-4-1	1	11,709,531	-1	ctta**T**tttt
30-4-1	3	11,616,755	-1	tttt**C**cgac
30-7-1	1	2,312,788	-1	cttg**T**ttta
30-7-1	1	25,449,577	-1	cata**G**tatt

Deleted bases are shown in bold. Homopolymeric sequences are underlined. Polynucleotide repeats are boxed. -1 mutations expected to be induced by 2-bp deletion followed by 1-bp insertion are not included.

Deletions of ≥2 bp identified in dry-seed irradiation.

Sample	Chr	Position	Type of mutation	Sequence	Size (bp)
125-10-5	1	25,115,764	Del_≥2 bp	ttca**CT**aact	-2
125-10-5	3	16,755,265	Del_≥2 bp	gaaa**TC**tcaa	-2
125-12-1	2	7,156,412	Del_≥2 bp	taat**AA**agtt	-2
125-12-1	3	21,581,283	Del_≥2 bp	gtgg**AT**taat	-2
125-2-2	2	14,419,918	Del_≥2 bp	tgag**TT**tttt	-2
125-2-2	4	10,723,555	Del_≥2 bp	acca**GT**atgc	-2
125-5-1	3	5,294,313	Del_≥2 bp	tcaa**AC**acat	-2
125-6-1	3	17,786,949	Del_≥2 bp	agga**CC**aaag	-2
175-1-4	5	16,344,361	Del_≥2 bp	acag**AA**caca	-2
175-2-4	5	24,286,276	Del_≥2 bp	cagg**TA**tata	-2
175-5-1	1	5,166,926	Del_≥2 bp	agtt**CC**ttcc	-2
175-5-1	2	395,716	Del_≥2 bp	atct**AG**agat	-2
175-5-1	3	7,522,459	Del_≥2 bp	ttca**AT**atct	-2
175-5-1	5	13,307,876	Del_≥2 bp	ctgt**TC**acgc	-2
175-6-1	1	4,530,659	Del_≥2 bp	ctca**GC**taac	-2
175-6-1	1	10,940,869	Del_≥2 bp	gagc**GT**catt	-2
175-6-1	4	5,470,763	Del_≥2 bp	tccg**AA**acaa	-2
125-10-5	4	15,014,172	Del_≥2 bp	aatt**AAA**aaag	-3
125-12-1	4	10,472,631	Del_≥2 bp	gatg**CTC**ctcc	-3
125-2-2	4	17,312,251	Del_≥2 bp	attt**TAA**taga	-3
125-5-1	2	13,824,297	Del_≥2 bp	ggtt**ATG**tttt	-3
125-5-1	3	4,153,732	Del_≥2 bp	cttg**CTA**ctca	-3
125-5-1	5	1,091,474	Del_≥2 bp	actg**CTT**cttg	-3
125-5-1	5	16,579,478	Del_≥2 bp	gacc**AAT**gtac	-3
125-6-1	3	8,041,753	Del_≥2 bp	tatt**TTG**aaaa	-3
125-6-1	4	6,696,328	Del_≥2 bp	aatg**TAA**tagc	-3
175-12-1	3	11,406,835	Del_≥2 bp	agaa**TTG**cctt	-3
175-12-1	5	6,481,553	Del_≥2 bp	gcaa**ATT**aatg	-3
175-1-4	2	2,788,013	Del_≥2 bp	acta**AAC**ctac	-3
175-1-4	5	16,617,906	Del_≥2 bp	ctgg**TAA**gcat	-3
175-2-4	4	4,274,350	Del_≥2 bp	tcta**CAT**catc	-3
175-4-1	1	22,115,903	Del_≥2 bp	ccta**ACT**acat	-3
175-4-1	4	616,130	Del_≥2 bp	atat**GAA**gaaa	-3
175-5-1	4	4,180,450	Del_≥2 bp	atat**GCA**atga	-3
175-6-1	1	5,341,405	Del_≥2 bp	tcaa**TGT**tgtt	-3
125-5-1	5	1,560,738	Del_≥2 bp	ttat**AAGA**aaga	-4
175-12-1	5	18,900,692	Del_≥2 bp	agac**CAAA**aaaa	-4
175-1-4	1	20,186,405	Del_≥2 bp	acca**AAAC**cact	-4
175-2-4	1	2,878,334	Del_≥2 bp	tata**GTTT**gatt	-4
175-4-1	3	9,031,896	Del_≥2 bp	ttag**TTAT**ttat	-4
175-4-1	4	15,364,201	Del_≥2 bp	gagg**TAAC**taca	-4
175-5-1	1	17,303,879	Del_≥2 bp	agag**GTAC**gtaa	-4
175-5-1	1	27,457,660	Del_≥2 bp	tgtc**CTAA**ctaa	-4

175-6-1	4	18,553,820	Del_≥2 bp	gagt**TTCC**ttca	-4
125-2-2	1	9,124,997	Del_≥2 bp	cgat**AATCA**gaat	-5
125-2-2	4	14,147,272	Del_≥2 bp	atct**TCATC**ttca	-5
125-4-1	5	16,424,864	Del_≥2 bp	tttc**CGGCT**aatt	-5
125-5-1	1	6,253,689	Del_≥2 bp	atga**ATTAG**gtgc	-5
125-5-1	1	26,920,198	Del_≥2 bp	ttaa**GAGAT**ggta	-5
125-5-1	5	25,068,035	Del_≥2 bp	ttca**AATCG**actc	-5
175-12-1	5	11,147,410	Del_≥2 bp	ttcc**TAACA**agca	-5
175-12-1	5	25,748,360	Del_≥2 bp	tgac**TAATT**tttt	-5
175-2-4	3	12,794,444	Del_≥2 bp	aatc**TTTTT**ttta	-5
175-4-1	4	13,728,384	Del_≥2 bp	atcc**ACAAA**acaa	-5
125-12-1	2	7,709,941	Del_≥2 bp	tact**ATGAAC**atta	-6
125-2-2	5	18,767,398	Del_≥2 bp	gtga**TGGTTG**tgac	-6
125-4-1	5	15,405,223	Del_≥2 bp	tttg**CTAAAC**cttg	-6
125-5-1	1	26,072,351	Del_≥2 bp	taag**AGAGGA**caag	-6
125-5-1	2	6,937,742	Del_≥2 bp	gtca**TGCTTC**tcta	-6
125-6-1	3	15,878,581	Del_≥2 bp	gaaa**AGGGAG**agat	-6
175-5-1	2	16,163,504	Del_≥2 bp	gaag**GCAAAC**tagg	-6
175-6-1	1	25,253,238	Del_≥2 bp	cacc**ACCGTT**aaga	-6
175-6-1	5	9,293,322	Del_≥2 bp	atgc**AGGCGA**ttta	-6
125-5-1	1	28,799,771	Del_≥2 bp	gaaa**TGATACC**tgag	-7
125-6-1	5	25,905,384	Del_≥2 bp	tgta**TTTGAAT**caat	-7
175-12-1	3	15,239,723	Del_≥2 bp	ccta**TAAATAC**tgaa	-7
175-1-4	4	4,704,733	Del_≥2 bp	tccc**TAAGCGA**gttc	-7
125-2-2	1	24,726,936	Del_≥2 bp	ttat**TACAGATC**taca	-8
125-4-1	3	11,165,929	Del_≥2 bp	agct**ACAAAATC**acaa	-8
125-5-1	3	9,239,983	Del_≥2 bp	ttac**TCCAAGTG**taga	-8
125-5-1	4	18,242,556	Del_≥2 bp	gcag**AATCAGGT**tctg	-8
125-6-1	5	13,796,775	Del_≥2 bp	gcta**AACTAAA**Accca	-8
175-2-4	2	12,367,411	Del_≥2 bp	tgga**ACTCTTTT**tttt	-8
175-2-4	5	18,926,429	Del_≥2 bp	atta**GATAACAT**atag	-8
175-4-1	1	5,652,997	Del_≥2 bp	tgct**TTGTTTGG**acaa	-8
175-5-1	2	10,888,982	Del_≥2 bp	atgt**ATTGTTAG**aaat	-8
175-5-1	5	26,591,541	Del_≥2 bp	tttg**GCTCACGA**gatg	-8
125-12-1	1	824,389	Del_≥2 bp	agac**GTCTAATTT**tttt	-9
125-12-1	2	2,546,303	Del_≥2 bp	aatg**ATGGCGACA**atga	-9
125-6-1	2	13,026,798	Del_≥2 bp	tagt**TTTGTTTGG**tttt	-9
125-10-5	5	15,422,503	Del_≥2 bp	gaaa**AAAA…GAAT**cacg	-12
125-6-1	1	4,623,134	Del_≥2 bp	tgtt**TCTA…AACA**tcaa	-12
175-2-4	3	18,497,940	Del_≥2 bp	gatg**TGAA…GTAA**tgaa	-12
175-6-1	1	18,637,436	Del_≥2 bp	aatg**TTGT…ACCC**ttgt	-12
175-6-1	1	24,901,914	Del_≥2 bp	ttaa**ATGA…GATG**ataa	-12
125-5-1	1	11,886,904	Del_≥2 bp	ctag**AGAA…TGCT**agaa	-13
175-2-4	1	11,770,737	Del_≥2 bp	actc**CAAC…TTC**Catac	-13
175-5-1	1	5,668,629	Del_≥2 bp	tctg**ATCG…GGAA**atca	-13

175-5-1	1	12,144,842	Del_≥2 bp	ttca**AGCT…GTTC**tgtt	-13
175-6-1	4	12,953,725	Del_≥2 bp	tggc**TCAA…ACGT**tagc	-13
125-12-1	5	3,487,785	Del_≥2 bp	gaat**ATAA…CAGG**aaga	-14
175-5-1	2	16,319,558	Del_≥2 bp	gggt**GTAA…ATAA**aata	-14
175-2-4	4	12,174,699	Del_≥2 bp	acag**AGAT…TAAA**actc	-16
175-5-1	3	1,457,700	Del_≥2 bp	cttg**TCAA…ATGA**tcaa	-16
175-5-1	4	11,621,149	Del_≥2 bp	aaat**TTAC…AAAA**ttac	-16
125-12-1	5	17,528,084	Del_≥2 bp	gccg**GAGG…GACC**tagt	-17
125-2-2	5	20,891,510	Del_≥2 bp	agtg**ATTT…GTAC**atct	-17
125-5-1	2	12,559,488	Del_≥2 bp	gagt**TCAT…CTAC**tcat	-17
175-4-1	3	8,187,494	Del_≥2 bp	atat**AGTT…AAAA**gctt	-18
175-6-1	1	6,417,811	Del_≥2 bp	caac**ACTT…CAAG**aatt	-18
125-10-5	2	6,248,477	Del_≥2 bp	ggag**TTTT…CTCT**ttgt	-22
125-4-1	1	10,335,193	Del_≥2 bp	gcaa**AGAG…ACAG**aga	-23
125-6-1	5	24,360,523	Del_≥2 bp	tagt**AAAG…ACTA**accg	-23
175-6-1	4	11,821,711	Del_≥2 bp	tcga**ATGT…TCGC**atgc	-24
175-4-1	3	18,753,702	Del_≥2 bp	atcc**AGTT…CAAA**agtt	-25
125-12-1	2	364,655	Del_≥2 bp	tgaa**ACCA…AAAT**atca	-26
125-2-2	2	14,816,650	Del_≥2 bp	cagt**CAGC…TGAC**ttct	-26
125-2-2	4	8,128,297	Del_≥2 bp	ctca**GTTG…GAAG**gtaa	-26
175-6-1	2	3,849,780	Del_≥2 bp	cttg**TTAA…CTAA**tgac	-28
175-4-1	5	20,924,511	Del_≥2 bp	attg**GTCC…TTAA**gttc	-29
175-1-4	1	8,909,976	Del_≥2 bp	actg**TCGG…AATT**tcgg	-30
125-4-1	5	24,449,837	Del_≥2 bp	atgc**GAAT…AAGT**gaat	-32
125-5-1	3	5,002,764	Del_≥2 bp	tcct**TTGG…TGAT**tgat	-32
175-2-4	3	13,531,918	Del_≥2 bp	gaac**CGTA…CTGT**cgtt	-32
125-2-2	4	9,618,651	Del_≥2 bp	ataa**GAAG…GCTC**ttac	-36
175-1-4	5	26,505,636	Del_≥2 bp	cggt**AGAA…CGGC**agg	-36
125-5-1	2	14,944,754	Del_≥2 bp	tccc**TTTT…TAGC**aaac	-37
175-12-1	3	4,087,237	Del_≥2 bp	tgta**AGCT…TGTT**gaag	-37
125-2-2	5	296,639	Del_≥2 bp	actc**CGTT…CAGA**gtcg	-44
175-2-4	5	2,024,079	Del_≥2 bp	gcat**TCGA…CCAG**gatt	-45
125-4-1	5	4,935,416	Del_≥2 bp	aact**TAAG…TACA**aact	-52
175-2-4	1	17,258,954	Del_≥2 bp	ggca**GCGT…AGCA**ttac	-67
125-10-5	4	11,649,411	Del_≥2 bp	cttt**CTTT…GTAA**ttgt	-144
175-5-1	3	17,667,864	Del_≥2 bp	ttca**TCTC…TGAT**ctta	-166
125-10-5	1	29,787,264	Del_≥2 bp	atat**AGAA…CTAC**gagc	-308
125-5-1	4	4,902,653	Del_≥2 bp	attc**ACTA…TAGA**acgg	-1,264
125-12-1	2	19,344,169	Del_≥2 bp	agat**ACAG…ACAT**tttt	-6,313
125-6-1	2	6,349,896	Del_≥2 bp	tata**TTGG…TCCG**agag	-245,416
175-4-1	5	15,108,168	Del_≥2 bp	atct**TCTC…AAAA**acta	-281,933

Deleted sequences are shown in bold. Homopolymeric sequences are underlined. Polynucleotide repeats or microhomologous sequences found in rejoined ends are boxed. Nine mutations accompanied by an insertion of unknown sequence are not included.

Deletions of ≥2 bp identified in seedling irradiation.

Sample	Chr	Position	Type of mutation	Sequence	Size (bp)
20-11-2	4	17,731,957	Del_≥2 bp	tatt**AA**aaaa	-2
30-3-4	5	578,928	Del_≥2 bp	tcac**AA**aatt	-2
20-2-3	1	28,183,231	Del_≥2 bp	agaa**TCC**tcct	-3
20-5-3	1	21,625,965	Del_≥2 bp	ggac**GAA**gaag	-3
30-4-1	3	23,420,715	Del_≥2 bp	aaga**TCT**tctt	-3
30-7-1	5	16,571,621	Del_≥2 bp	tact**CAA**ggtg	-3
30-7-1	3	6,329,883	Del_≥2 bp	gatg**TTTC**tggt	-4
20-2-3	5	6,014,221	Del_≥2 bp	caca**GAAAC**atat	-5
30-4-1	1	10,341,117	Del_≥2 bp	ggtt**TATAG**tata	-5
20-3-1	4	3,447,499	Del_≥2 bp	ctca**TCACTC**taaa	-6
30-3-4	3	84,833	Del_≥2 bp	aaca**TAGCTT**tagg	-6
30-4-1	1	20,382,260	Del_≥2 bp	tcca**GGACCG**ccgg	-6
30-7-1	5	8,938,331	Del_≥2 bp	acga**GAAGGG**tttg	-6
20-1-2	2	198,917	Del_≥2 bp	tact**ACCACAAC**acta	-8
30-11-2	1	6,542,443	Del_≥2 bp	tatt**TATAAGTC**tata	-8
30-6-2	1	7,378,262	Del_≥2 bp	caca**TGACTGAT**tacc	-8
30-7-1	3	1,752,879	Del_≥2 bp	gcat**CTTCTCTA**atga	-8
20-11-2	2	4,042,421	Del_≥2 bp	ctat**AATTGGTCC**tgtg	-9
20-11-2	3	20,481,257	Del_≥2 bp	agat**CATACACAA**catt	-9
30-4-1	3	19,437,059	Del_≥2 bp	aaat**AAAACTTTCA**aaat	-10
20-5-3	4	17,382,806	Del_≥2 bp	aaat**ATAA…TTGG**aaag	-12
20-1-2	3	18,528,885	Del_≥2 bp	aatg**TTGG…TTGC**ttga	-13
30-7-1	4	7,410,976	Del_≥2 bp	tact**CCAT…TTCA**ccac	-13
30-7-1	3	22,551,700	Del_≥2 bp	gaaa**ATGT…GGTC**atgt	-14
30-7-1	2	14,524,698	Del_≥2 bp	ttca**AGTT…TCAT**atgt	-15
20-1-2	1	12,363,887	Del_≥2 bp	ctct**CATT…TAGG**acaa	-19
20-11-2	3	18,920,796	Del_≥2 bp	cagt**CCCT…AGGA**gagt	-21
20-11-2	5	1,079,961	Del_≥2 bp	aatc**TTAT…CTAA**ttat	-24
20-2-3	2	806,206	Del_≥2 bp	tggg**GAAC…CTGA**gtta	-24
20-3-1	5	18,522,272	Del_≥2 bp	aatt**ATCT…AAGA**tttt	-24
30-7-1	3	15,523,392	Del_≥2 bp	tggc**TCAG…GAGC**tgtt	-24
20-1-2	4	18,210,058	Del_≥2 bp	caca**GGTA…CTGTT**ggaa	-26
20-11-2	4	11,260,291	Del_≥2 bp	gtca**TCGA…GCCC**tccc	-27
20-1-2	5	8,612,292	Del_≥2 bp	aacc**AAAC…TATA**aaat	-33
30-2-1	2	1,093,918	Del_≥2 bp	actc**TGTT…ATGT**tgtg	-33
30-11-2	4	6,596,785	Del_≥2 bp	cggt**AGAA…CACT**gtcc	-36
20-4-4	5	8,315,271	Del_≥2 bp	aggg**AATG…TGAG**aggg	-41
20-1-2	3	5,738,697	Del_≥2 bp	gaga**GCTA…GGAG**gctg	-45
20-5-3	4	14,028,056	Del_≥2 bp	ttgg**GTTG…TTGG**aaca	-57
20-5-3	5	17,811,077	Del_≥2 bp	tgat**GGGG…CTAT**tatg	-141

20-5-3	3	1,671,663	Del_≥2 bp	actg**GCTC…CCAA**gcat	-153
20-3-1	2	7,140,512	Del_≥2 bp	cttg**AACC…GTAG**gaga	-480
30-2-1	5	4,243,291	Del_≥2 bp	tccg**TCAC…ATAG**agag	-1,441
20-2-3	1	17,130,087	Del_≥2 bp	ttga**GTAC…TAGA**agaa	-1,901

Deleted sequences are shown in bold. Homopolymeric sequences are underlined. Polynucleotide repeats or microhomologous sequences found in rejoined ends are boxed. Four mutations accompanied by an insertion of unknown sequence are not included.

Single base insertions identified in dry-seed irradiation.

Sample	Chr	Position	Type of mutation	Sequence	Inserted nucleotide
125-12-1	4	15,342,414	+1	tttt*aaaa	A
125-2-2	1	9,148,100	+1	aata*aata	T
125-2-2	1	11,733,176	+1	acct*ggag	G
125-2-2	1	23,705,214	+1	gtag*catt	T
125-2-2	3	15,023,913	+1	tgga*tttt	T
125-2-2	5	23,626,714	+1	attg*aaat	A
125-6-1	2	18,374,032	+1	ttca*taac	T
125-6-1	5	21,016,447	+1	tttt*aaaa	A
175-12-1	4	3,860,342	+1	atta*tttt	T
175-12-1	4	13,973,558	+1	aagc*ttgc	T
175-2-4	3	9,305,559	+1	ttta*taaa	A
175-2-4	3	21,633,156	+1	cttc*ttat	T
175-2-4	4	16,779,820	+1	ctac*aaaa	A
175-4-1	1	16,252,297	+1	ttgc*aatt	A

Asterisks indicate insertion position. Homopolymeric sequences are underlined. Polynucleotide repeats are boxed. Two mutations expected to be induced by “3-bp deletion followed by 4-bp insertion” and “2-bp deletion followed by 3-bp insertion” are not included.

Single base insertions identified in seedling irradiation.

Sample	Chr	Position	Type of mutation	Sequence	Inserted nucleotide
20-11-2	1	26,986,773	+1	ggga*ttcg	T
20-4-4	2	7,204,908	+1	taat*aaaa	A
30-11-2	1	18,032,053	+1	acag*aaaa	A
30-7-1	1	4,861,400	+1	caac*aaaa	A

Asterisk indicates insertion position. Homopolymeric sequences are underlined. Polynucleotide repeats are boxed.

Insertions of ≥2bp identified in dry-seed irradiation.

Sample	Chr	Position	Type of mutation	Sequence	Inserted nucleotide	Size (bp)
125-4-1	1	26,453,736	Ins_≥2 bp	tctg*atat	AT	+2
125-4-1	4	15,565,948	Ins_≥2 bp	aaag*atat	AT	+2
125-6-1	5	23,475,568	Ins_≥2 bp	ataa*agag	AG	+2
175-1-4	3	1,064,359	Ins_≥2 bp	tagc*atat	AT	+2
175-2-4	4	1,640,569	Ins_≥2 bp	gaac*tttt	TT	+2
175-2-4	4	14,290,505	Ins_≥2 bp	gtgt*tgtg	TG	+2
175-4-1	1	14,482,543	Ins_≥2 bp	agtc*tgaa	TT	+2
175-6-1	4	12,433,190	Ins_≥2 bp	ggtc*gctg	GCT	+3
125-4-1	1	18,999,155	Ins_≥2 bp	cctt*tttc	TTTC	+4
175-1-4	2	17,134,939	Ins_≥2 bp	cttg*cgaa	CGAAT	+5
175-12-1	2	9,454,654	Ins_≥2 bp	ttca*aatt	AATTTTCACT	+10
175-5-1	2	19,208,090	Ins_≥2 bp	taaa*aatt	ATAA…ATGT	+17
125-12-1	1	20,607,208	Ins_≥2 bp	cgag*tatt	TTTT…CAAA	+27

Asterisk indicates insertion position. Homopolymeric sequences are underlined. Polynucleotide repeats or microhomologous sequences found in rejoined ends are boxed.

Insertions of ≥2bp identified in seedling irradiation.

Sample	Chr	Position	Type of mutation	Sequence	Inserted nucleotide	Size (bp)
20-1-2	3	23,378,897	Ins_≥2 bp	ggat*taag	TAGCC	+5
30-7-1	3	1,954,024	Ins_≥2 bp	ctct*tttt	TTTTTG	+5
30-3-4	5	9,561,866	Ins_≥2 bp	tctg*tgtt	TGTT…TTCA	+12
30-11-2	1	8,024,381	Ins_≥2 bp	aaaa*cgaa	CGTA…AATT	+47

Asterisk indicates insertion position. Homopolymeric sequences are underlined. Polynucleotide repeats or microhomologous sequences found in rejoined ends are boxed.

Table S6. Details of complex-type mutations.

Complex-type mutations identified in dry-seed irradiation.

Sample	Chr	Position	Type of mutation	Original sequence	Altered sequence	Summary
125-10-5	2	5,641,286	Complex	tatt**CC**attt	tatt**AT**attt	SBS×2
125-12-1	5	1,553,890	Complex	gaaa**AG**taaa	gaaa**TT**taaa	SBS×2
125-2-2	4	6,671,908	Complex	tggg**GT**cgtg	tggg**AA**cgtg	SBS×2
125-2-2	4	11,005,321	Complex	tgct**GT**caaa	tgct**AA**caaa	SBS×2
125-2-2	5	21,587,693	Complex	ttat**CT**tcat	ttat**TC**tcat	SBS×2
125-4-1	2	2,827,384	Complex	ttgg**AG**agac	ttgg**GA**agac	SBS×2
125-5-1	5	20,905,119	Complex	ataa**GG**attt	ataa**AA**attt	SBS×2
125-5-1	5	22,060,358	Complex	ctaa**CC**aaaa	ctaa**AA**aaaa	SBS×2
175-2-4	3	21,185,003	Complex	gaag**CC**taga	gaag**AA**taga	SBS×2
175-4-1	4	5,247,540	Complex	gcgc**GA**cgta	gcgc**AT**cgta	SBS×2
175-5-1	5	4,537,230	Complex	atgt**AC**tcta	atgt**TT**tcta	SBS×2
175-6-1	1	10,292,187	Complex	gagt**TT**gcca	gagt**AA**gcca	SBS×2
175-6-1	4	2,659,973	Complex	tttc**AT**ataa	tttc**GA**ataa	SBS×2
175-6-1	5	5,995,045	Complex	cgaa**AG**attc	cgaa**TT**attc	SBS×2
125-4-1	5	16,591,716	Complex	gcta**A**t**T**tcgt	gcta**T**t**C**tcgt	SBS×2 (1 bp gap)
125-6-1	3	15,886,369	Complex	attg**C**a**C**atat	attg**A**a**T**atat	SBS×2 (1 bp gap)
175-1-4	5	8,145,982	Complex	attt**T**a**T**tagg	attt**A**a**C**tagg	SBS×2 (1 bp gap)
175-5-1	4	3,276,508	Complex	caat**G**g**C**tgca	caat**T**g**G**tgca	SBS×2 (1 bp gap)
125-12-1	2	13,864,099	Complex	cacc**A**cc**A**ccgt	cacc**C**cc**C**ccgt	SBS×2 (2 bp gap)
125-5-1	5	14,691,336	Complex	gcaa**T**aaa**G**attc	gcaa**A**aaa**C**attc	SBS×2 (3 bp gap)
125-2-2	1	24,907,911	Complex	aaat**T**acgg**A**ttta	aaat**C**acgg**G**ttta	SBS×2 (4 bp gap)
175-4-1	1	5,773,394	Complex	ccta**G**ttattt**G**tttt	ccta**A**ttattt**A**tttt	SBS×2 (6 bp gap)
175-1-4	5	16,351,557	Complex	caatcg**A**tgag	caat**CCA**cg**T**tgag	+3 bp, SBS (2 bp gap)
175-6-1	1	24,542,359	Complex	ccat**G**gggccg**T**ttca	ccatgggccg**C**ttca	-1 bp, SBS (6 bp gap)
125-5-1	4	10,605,193	Complex	taaa**AAGGTGAA**ac**T**aaaa	taaa**TTC**ac**C**aaaa	-8+3 bp, SBS (2 bp gap)
175-6-1	2	12,287,890	Complex	catc**CA**ctga**TCCTAC**tttc	catcctga**AA**tttc	-2 bp, -6+2 bp (4 bp gap)

175-12-1	1	19,191,737	Complex	catg**AATTT**ctg**CTTC**ttct	catg**GAA**ctgttct	-5+3 bp, -4 bp (3 bp gap)
175-6-1	1	27,974,164	Complex	cctt**CTTTTTTTTAAA**aaa**A**atat	cctt**AT**aaa**C**atat	-12+2 bp, SBS (3 bp gap)
175-6-1	3	20,597,318	Complex	gata**GGCA…ATCCAA**acaaa**A**caat	gata**AC**acaaa**T**caat	-26+2 bp, SBS (5 bp gap)
175-6-1	1	24,542,344	Complex	cttt**T**tgatacc**T**tgaagtga**A**gggt	cttt**C**tgat**GA**acc**C**tgaagtga**C**gggt	More than 3 changes

Altered sequences are shown in bold.

Complex-type mutations identified in seedling irradiation.

Sample	Chr	Position	Type of mutation	Original sequence	Altered sequence	Summary
20-2-3	2	10,262,502	Complex	gata**AA**gtat	gata**GC**gtat	SBS×2
20-2-3	5	1,099,628	Complex	tgac**CA**ctta	tgac**TT**ctta	SBS×2
20-3-1	3	7,693,524	Complex	ttct**TC**ctca	ttct**CT**ctca	SBS×2
20-4-4	5	6,317,860	Complex	gtat**TT**ttac	gtat**AA**ttac	SBS×2
20-5-3	3	8,579,840	Complex	aatc**TA**tttc	aatc**AT**tttc	SBS×2
30-11-2	4	6,986,614	Complex	agtt**TG**aaaa	agtt**AA**aaaa	SBS×2
30-4-1	2	6,908,771	Complex	aaga**GC**tgac	aaga**AA**tgac	SBS×2
30-7-1	3	16,961,207	Complex	cagt**GG**agcc	cagt**CA**agcc	SBS×2
20-11-2	1	20,029,121	Complex	agaa**GCA**tgtt	agaa**AGT**tgtt	SBS×3
20-5-3	3	2,162,882	Complex	atgc**T**a**G**gata	atgc**C**a**T**gata	SBS×2 (1 bp gap)
20-1-2	3	14,493,990	Complex	aaaa**G**at**C**tctt	aaaa**T**at**T**tctt	SBS×2 (2 bp gap)
30-3-4	3	3,237,415	Complex	ttga**A**ag**G**acca	ttga**T**ag**T**acca	SBS×2 (2 bp gap)
20-1-2	3	19,169,271	Complex	caga**A**gaa**G**atct	caga**T**gaa**C**atct	SBS×2 (3 bp gap)
30-4-1	2	5,670,355	Complex	acct**C**cat**A**tcaa	acct**T**cat**G**tcaa	SBS×2 (3 bp gap)
20-2-3	3	183,269	Complex	cttg**T**tgaga**T**aaat	cttg**G**tgaga**G**aaat	SBS×2 (5 bp gap)
20-1-2	2	17,239,430	Complex	ataa**AATTG**tta**T**taaa	ataa**T**tta**ACAAA**taaa	-5+1 bp, -1+5 bp (3 bp gap)
30-6-2	1	3,859,020	Complex	ggctag**T**ctat	ggct**TTATCCGAAATG**ag**AG**ctat	+12 bp, -1+2 bp (2 bp gap)
30-6-2	1	12,448,959	Complex	tcta**C**gtatcgtg	tcta**T**gtat**ATGA…CATG**cgtg	SBS, +97 bp (4 bp gap)
30-6-2	2	17,255,001	Complex	aatt**TAGA**tatc**A**aatc	aatttatc**G**aatc	-4 bp, SBS (4 bp gap)
30-11-2	5	11,984,996	Complex	gata**TTCTCGGATTTT**at**T**gagg	gataat**G**gagg	-12 bp, SBS (2 bp gap)

30-2-1	1	3,997,615	Complex	agaa**GATG…TGTG**ctcgtt	agaact**GGAAAA**cgtt	-36 bp, +6 bp (2 bp gap)
20-5-3	5	5,369,001	Complex	atgc**TTG**agagaa**GTTT…TTT**gttg	atgc**ATAA**agagaagttg	-3+4 bp, -24 bp (6 bp gap)
20-11-2	1	24,023,841	Complex	ctcc**TTTTCTG**ct**G**a**C**cactcttgt	ctcc**AGT**ct**A**a**G**ca**TTACT**ctc**CGAAATCTGG**ttgt	More than 3 changes
20-11-2	3	2,999,203	Complex	attt**TCAAAACCCTAAGC**t**A**at**T**tttt**A**aa**AAATTTGTATG**aa**TT**taga	atttt**T**at**A**tttt**C**aaaa**CG**taga	More than 3 changes
20-1-2	2	16,832,880	Complex	gggt**G**agaa**AG**aa**T**aa**CCT**actt	gggtagaa**GC**aa**G**aaactt	More than 3 changes
30-7-1	5	26,821,710	Complex	actc**T**agt**A**tca**A**tgc**TT**aaga	actc**C**agt**T**t**TTTC**ca**G**t**G**gcaaga	More than 3 changes

Altered sequences are shown in bold.

Table S7. Number of mutation events that resulted in non-synonymous amino acid changes in protein coding genes.

Dry-seed 125 Gy							
Sample	125-10-5	125-12-1	125-2-2	125-4-1	125-5-1	125-6-1	Total
Homo	0	1	4	0	4	2	11 (1.8 ± 0.7)
Hetero	7	6	10	5	8	2	38
Total	7	7	14	5	12	4	49 (6.3 ± 1.1)
Dry-seed 175 Gy							
Sample	175-1-4	175-12-1	175-2-4	175-4-1	175-5-1	175-6-1	Total
Homo	4	1	1	5	7	2	20 (3.3 ± 1.0)
Hetero	2	2	10	6	6	13	39
Total	6	3	11	11	13	15	59 (6.5 ± 1.8)
Seedling 20 Gy							
Sample	20-11-2	20-1-2	20-2-3	20-3-1	20-4-4	20-5-3	Total
Homo	2	1	0	3	1	0	7 (1.2 ± 0.5)
Hetero	3	6	4	0	0	14	27
Total	5	7	4	3	1	14	34 (4.5 ± 2.1)
Seedling 30 Gy							
Sample	30-11-2	30-2-1	30-3-4	30-4-1	30-6-2	30-7-1	Total
Homo	0	1	1	1	0	0	3 (0.5 ± 0.2)
Hetero	0	4	2	3	1	7	17
Total	0	5	3	4	1	7	20 (2.8 ± 1.0)

Transposable elements, pseudogenes, and structural variations are not included.

Numbers in parentheses represent mean ± standard error of 6 samples in each group.

* Ratio could not be calculated, because all identified mutations were heterozygous.

** Data from sample 30-7-1 were not included in calculation.
